# Benzofuran and Benzo[*b*]thiophene‐2‐Carboxamide Derivatives as Modulators of Amyloid Beta (Aβ42) Aggregation

**DOI:** 10.1002/cmdc.202400198

**Published:** 2024-09-30

**Authors:** Yusheng Zhao, Kartar Singh, Rahul Chowdary Karuturi, Ahmed A. Hefny, Arash Shakeri, Mike A. Beazely, Praveen P. N. Rao

**Affiliations:** ^1^ School of Pharmacy Health Sciences Campus University of Waterloo 200 University Avenue West, Waterloo N2L 3G1 Ontario Canada

**Keywords:** Alzheimer's disease, Amyloid-β beta aggregation, Small molecules, Aggregation inhibitors, Aggregation promoters

## Abstract

A group of *N*‐phenylbenzofuran‐2‐carboxamide and *N*‐phenylbenzo[*b*]thiophene‐2‐carboxamide derivatives were designed and synthesized as a novel class of Aβ42 aggregation modulators. In the thioflavin‐T based fluorescence aggregation kinetics study, compounds **4 a**, **4 b**, **5 a** and **5 b** possessing a methoxyphenol pharmacophore were able to demonstrate concentration dependent inhibition of Aβ42 aggregation with maximum inhibition of 54 % observed for compound **4 b**. In contrast, incorporation of a 4‐methoxyphenyl ring in compounds **4 d** and **5 d** led to a significant increase in Aβ42 fibrillogenesis demonstrating their ability to accelerate Aβ42 aggregation. Compound **4 d** exhibited 2.7‐fold increase in Aβ42 fibrillogenesis when tested at the maximum concentration of 25 μM. These results were further confirmed by electron microscopy studies which demonstrates the ability of compounds **4 a**, **4 b**, **4 d**, **5 a**, **5 b** and **5 d** to modulate Aβ42 fibrillogenesis. Compounds **5 a** and **5 b** provided significant neuroprotection to mouse hippocampal neuronal HT22 cells against Aβ42‐induced cytotoxicity. Molecular docking studies suggest that the orientation of the bicyclic aromatic rings (either benzofuran or benzo[*b*]thiophene) plays a major role in moderating their ability to either inhibit or accelerate Aβ42 aggregation. Our findings support the application of these novel derivatives as pharmacological tools to study the mechanisms of Aβ42 aggregation.

## Graphical abstract

A group of novel benzofuran and benzothiophene‐based small molecules were designed, synthesized and evaluated as amyloid‐beta aggregation modulators. The results obtained demonstrate that these class of molecules can be chemically modified to obtain either amyloid‐beta aggregation inhibitors or promoters. These studies demonstrate their potential application to develop novel diagnostics and therapeutics for Alzheimer's disease.

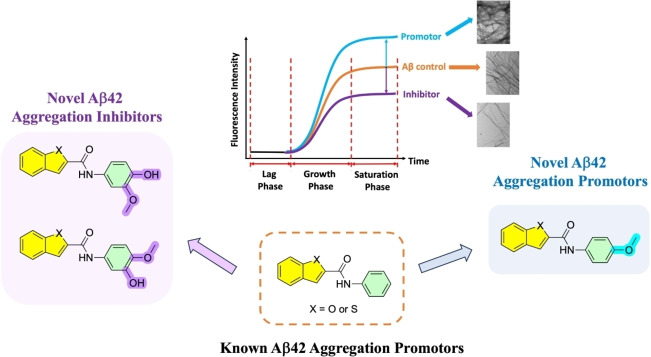

## Introduction

Alzheimer's disease (AD) is a complex neurodegenerative disorder characterized by the accumulation of amyloid beta (Aβ) plaques and neurofibrillary tangles (NFTs) consisting of the tau protein.[^1^, ^2^] The AD etiology is attributed to a number of mechanisms including the cholinergic dysfunction, amyloid cascade hypothesis, tau hyperphosphorylation, mitochondrial dysfunction and oxidative stress to mention a few.[^3^, ^4^, ^5^, ^6^, ^7^] Among them, amyloid cascade in AD continues to be a prominent focus of researchers worldwide, and lays the framework for much of our understanding on AD mechanisms and development of novel therapies.[^4^, ^8^, ^9^, ^10^] In fact, two anti‐amyloid based therapeutics were developed recently as novel treatment options for AD. The first one was aducanumab (Aduhelm®) which received accelerated approval by the US FDA and the second one was lecanumab (Leqembi®) which was launched in 2023.^[11]^ Other novel biological therapies which exhibit similar mechanism of action, are being developed.^[12]^ However, discovering novel small molecule based anti‐amyloid therapies is highly desirable as they can be administered orally, manufactured at a fraction of the cost compared to biological therapies, and are easy to store and transport.^[13]^

**Figure 1:**
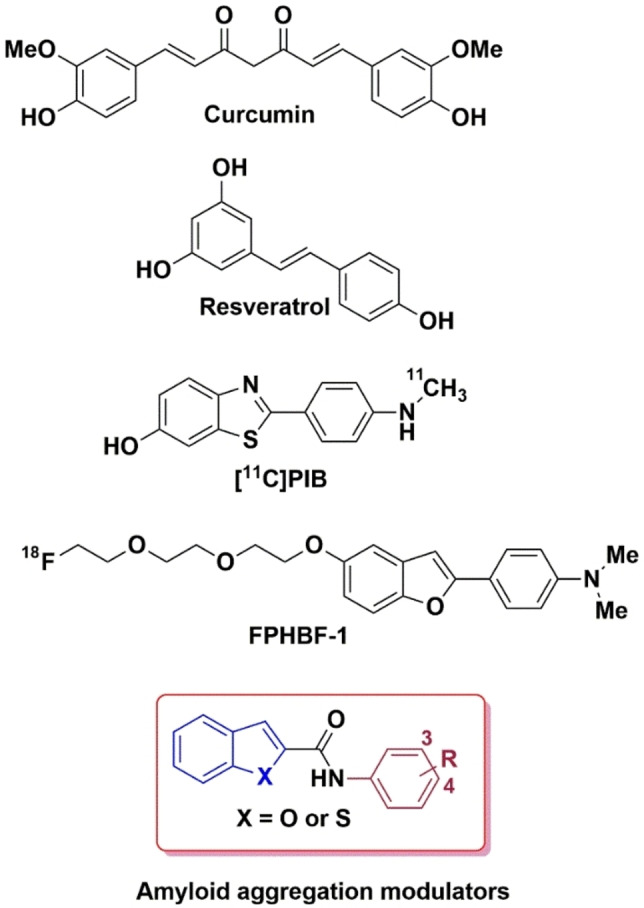
Chemical structures of amyloid aggregation modulators.

In the amyloid cascade, misprocessing of the amyloid precursor protein (APP) in the brain leads to the formation of Aβ peptides (Aβ40 and Aβ42) which undergo self‐assembly to form neurotoxic species such as oligomers, protofibrils and fibrils which promote neurodegeneration, and also trigger other secondary events to cause brain damage in AD.[^4^, ^8^] Understanding the molecular mechanisms of Aβ self‐assembly into toxic β‐sheet structures is a daunting task. In this regard, researchers were able to use structural biology methods to solve the 3D structures of Aβ fibrils[^14^, ^15^, ^16^] which has provided valuable insights on i) the mechanisms of Aβ self‐assembly; and ii) to design novel chemical tools and therapeutics to modulate the misfolding and aggregation pathway of Aβ. Small molecule based Aβ modulators represent an important class of compounds that have a wide range of application in AD research. For example, natural small molecule based compounds curcumin and resveratrol are inhibitors of Aβ aggregation with therapeutic potential to treat AD whereas small molecules possessing bicyclic aromatic rings such as benzothiazole and benzofuran rings have application as positron emission tomography (PET) imaging agents for detecting Aβ plaques in AD brain (Figure 1).[^17^, ^18^, ^19^, ^20^] The common structural feature present in these compounds is the presence of aromatic rings, and their planar conformation that allows them to interact and bind with the cross‐β‐sheet structures of Aβ aggregates.

While curcumin and resveratrol can prevent Aβ aggregation, they are not considered as good drug candidates due to a number of factors. For example, curcumin is considered as a pan‐assay interfering compound (PAINS) due to the presence of the α,β‐unsaturated Michael acceptor moiety that can react with biological thiols, and both curcumin and resveratrol exhibit poor pharmacokinetic properties.[^21^, ^22^, ^23^] In this regard, our previous work demonstrated that planar molecules containing either a bicyclic, aromatic benzofuran or benzothiophene rings linked by an amide bond to an unsubstituted aromatic ring, were able to modulate Aβ42 aggregation kinetics and mitigate its cytotoxicity in hippocampal HT22 neuronal cells.^[24]^ These compounds represent a novel class of chemical/pharmacological tools to study the mechanisms of Aβ42 aggregation. Based on these observations, we synthesized and evaluated a library of *N*‐(3,4‐substitutedphenyl)benzofuran‐2‐carboxamide and *N*‐(3,4‐substitutedphenyl)benzo[*b*]thiophene‐2‐carboxamide derivatives (Figure 1) as novel modulators of Aβ42 aggregation. In vitro Aβ42 aggregation kinetic studies were carried out using thioflavin T (ThT) based fluorescence spectroscopy, 8‐anilino‐1‐naphthalenesulfonic acid (ANS) fluorescent probe was used to determine the conformational changes of Aβ42 aggregates in the presence of benzofuran/benzothiophene derivatives, the morphology of Aβ42 aggregates in the presence of test compounds was studied by transmission electron microscopy (TEM), computational modeling studies were carried out to investigate the binding site location of ligands in Aβ42 assemblies and cell viability experiments were carried out to determine the effect of test compounds in preventing Aβ42 mediated toxicity in mouse hippocampal HT22 neuronal cells. These studies show that benzofuran and benzothiophene based carboxamides are able to bind and modulate the Aβ42 aggregation pathway and can rescue mouse hippocampal HT22 neuronal cells from Aβ42 mediated cytotoxicity.

## Results and Discussion

### Synthesis of Substituted Benzofuran and Bbenzo[*b*]thiophene Carboxamide Derivatives

**Scheme 1:**
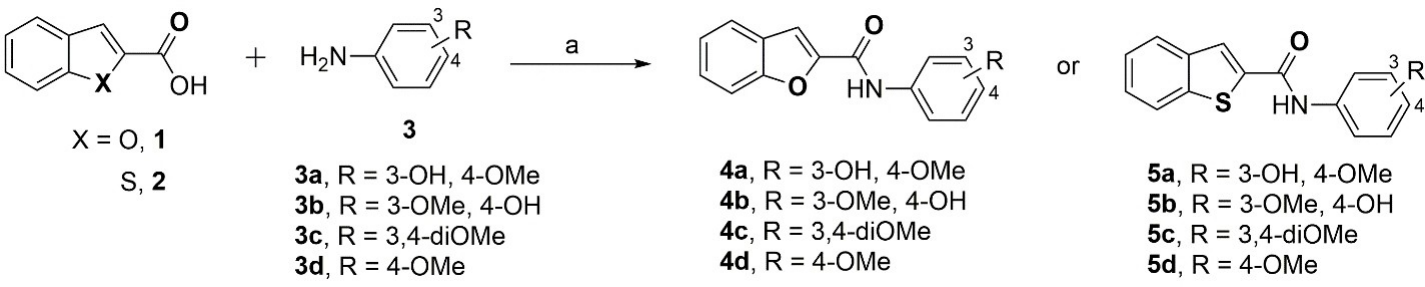
Synthesis of *N*‐(substitutedphenyl)benzofuran‐2‐carboxamide and benzo[*b*]thiophene‐2‐carboxamide derivatives **4 a**–**d** and **5 a**–**d**. Reagents and conditions: (a) EDC, HOBt, THF, room temperature, overnight.

The *N*‐(substitutedphenyl)benzofuran‐2‐carboxamide and *N*‐(substitutedphenyl)benzo[*b*]thiophene‐2‐carboxamide derivatives (**4 a**–**d** and **5 a**–**d**) were synthesized by one‐step direct coupling using 1‐ethyl‐3‐(3‐dimethylaminopropyl)carbodiimide (EDC), and hydroxybenzotriazole (HOBt) as summarized in Scheme 1.^[25]^ The corresponding acids, either benzofuran‐2‐carboxylic acid (**1**) or benzo[*b*]thiophene‐2‐carboxylic acid (**2**) were coupled with various aniline derivatives (**3 a**–**d**, Scheme 1) to give the final carboxamide derivatives **4 a**–**d** and **5 a**–**d** (Scheme 1). The final compounds were characterized by ^1^H and ^13^C NMR, liquid chromatography mass spectrometry (LCMS) and by high resolution mass spectrometry (HRMS) studies. Target compounds **4 a**–**d** and **5 a**–**d** were obtained in good yields (72.3–89.2 %).

### Thioflavin T (ThT) Based Aβ42 Aggregation Kinetics Assay

**Figure 2:**
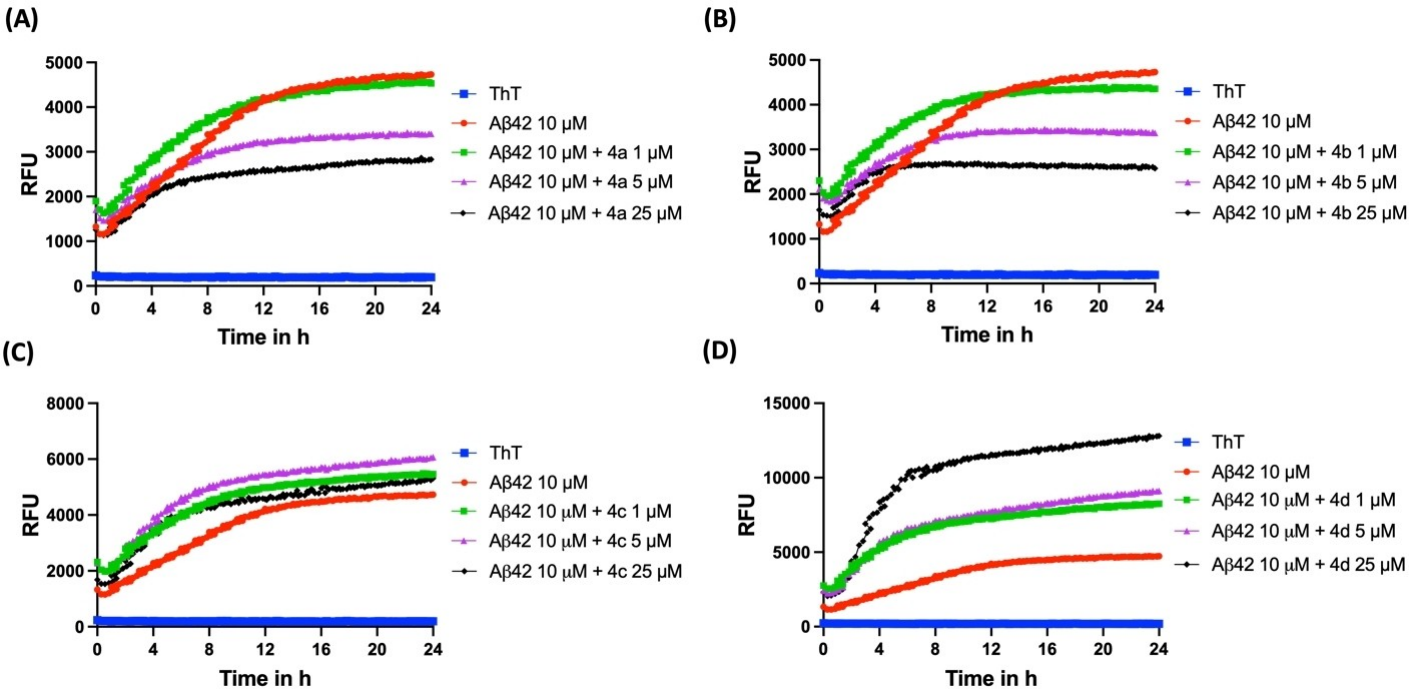
ThT‐based aggregation kinetic curve of Aβ42 (10 μM) in the presence and absence of **4 a**–**d** at 1, 5, 25 μM. (A) Aggregation kinetic curve for **4 a**. (B) Aggregation kinetic curve for **4 b**. (C) Aggregation kinetic curve for **4 c**. (D) Aggregation kinetic curve for **4 d**. The aggregation kinetics were monitored by ThT fluorescence RFUs at 440 nm excitation and 490 nm emission 24 h at 37 °C. Results are averages of three independent experiments (n=3).

The effect of compounds **4 a–d** and **5 a–d** to modulate the aggregation kinetics of Aβ42 was investigated using the thioflavin T (ThT) based fluorescence spectroscopy studies.[^26^, ^27^] The ThT dye shows a quantum shift in its fluorescence when it binds to β‐sheet structures and this method can be used to monitor the ability of test compounds to inhibit and or modulate Aβ42 aggregation.[^26^, ^27^] The ThT based Aβ42 aggregation studies were carried out over a period of 24 h. In the absence of test compounds, Aβ42 underwent rapid aggregation with a short lag phase (~2 h, Figure 2). This was followed by a drastic increase in the ThT fluorescence intensity (relative fluorescence units, RFU) in the next 10 h, indicating the formation of higher order fibrillar Aβ42 aggregates that are rich in β‐sheet content representing the growth phase. At the end of ~12 h, plateau phase was observed with the saturation of ThT fluorescence (RFUs) as seen in Figure 2. In the benzofuran‐2‐carboxamide series of compounds, the presence of a 2‐methoxyphenol moiety in compound **4 a** led to inhibition of Aβ42 aggregation (Figure 2). For example, in the presence of 1 μM of **4 a** (R=3‐OH, 4‐OMe) there was no major change in the ThT fluorescence intensity which indicates weak inhibition of Aβ42 aggregation (Panel A, Figure 2). However, when the concentration of **4 a** was increased to 5 μM and 25 μM respectively, there was a concentration dependent decline in the ThT fluorescence (RFUs) which demonstrates the ability of compound **4 a** to inhibit Aβ42 aggregation (Panel A, Figure 2). At 5 μM, compound **4 a** exhibited 21 % inhibition of Aβ42 aggregation at the end of 24 h time period. When the concentration was increased to 25 μM, compound **4 a** exhibited even better inhibition of Aβ42 aggregation (41 % inhibition). Evaluation of the corresponding regioisomer compound **4 b** (R=3‐OMe, 4‐OH), where the position of −OH and −OMe in the methoxyphenol pharmacophore was switched led to better inhibition of Aβ42 aggregation. Compound **4 b** exhibited a concentration dependent decline in the ThT fluorescence which demonstrates its ability to reduce Aβ42 aggregation (Panel B, Figure 2). At 5, 10 and 25 μM compound **4 b** showed 26 %, 47 % and 54 % inhibition Aβ42 aggregation respectively (Panel B, Figure 2). In further structure‐activity relationship (SAR) analysis, replacing the methoxyphenol moiety with a 3,4‐dimethoxyphenyl substituent was detrimental in retaining the anti‐aggregation properties of compounds **4 a** and **4 b**. Compound **4 c** (R=3,4‐diOMe) which possess the 3,4‐dimethoxyphenyl moiety did not modulate Aβ42 aggregation kinetics at the concentrations tested (1, 5 and 25 μM, Panel C, Figure 2), and did not exhibit any inhibition of Aβ42 aggregation. In contrast, when the 3,4‐dimethoxyphenyl moiety was replaced with a 4‐methoxyphenyl substituent, strikingly there was promotion of Aβ42 fibrillogenesis. For example, compound **4 d** (R=4‐OMe), which possess a 4‐methoxyphenyl moiety was able to promote Aβ42 fibrillogenesis at all the tested concentrations (Panel D, Figure 2). At 1 μM, compound **4 d** exhibited a dramatic and significant increase in ThT fluorescence intensity compared to Aβ42 alone treated wells demonstrating its ability to promote Aβ42 fibrillogenesis (Panel D, Figure 2). Compound **4 d** exhibited ~1.74‐fold promotion in Aβ42 fibrillogenesis at 1 μM, and with increasing concentrations, compound **4 d** exhibited further promotion of Aβ42 fibrillogenesis with 1.92 and 2.70‐fold increases seen at 5 and 25 μM respectively (Panel D, Figure 2). These observations are consistent with our previous work where the unsubstituted *N*‐phenylbenzofuran‐2‐carboxamide compound was able to cause significant promotion of Aβ42 fibrillogenesis.^[24]^

**Figure 3:**
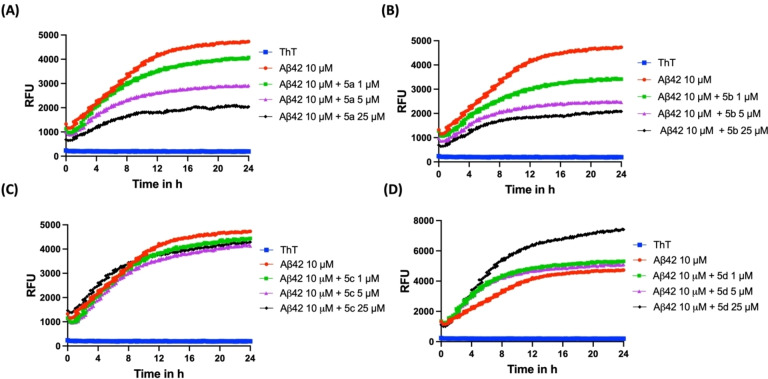
ThT‐based aggregation kinetic curve of Aβ42 (10 μM) in the presence and absence of **5 a**–**d** at 1, 5, 25 μM. (A) Aggregation kinetic curve for **5 a**. (B) Aggregation kinetic curve for **5 b**. (C) Aggregation kinetic curve for **5 c**. (D) Aggregation kinetic curve for **5 d**. The aggregation kinetics were monitored by ThT fluorescence RFUs at 440 nm excitation and 490 nm emission for 24 h at 37 °C. Results are averages of three independent experiments (n=3).

Evaluation of the benzo[*b*]thiophene‐2‐carboxamide series (**5 a**–**d**) in the ThT based fluorescence assay provided similar SAR as per the benzofuran‐2‐carboxamide series of compounds (**4 a**–**d**). When incubated with Aβ42, compound **5 a** (R=3‐OH, 4‐OMe) exhibited a concentration dependent decline in the ThT fluorescence during the 24 h time period (Panel A, Figure 3). There was ~6 %, 26 % and 40 % inhibition of Aβ42 aggregation in the presence of 1, 5 and 25 μM of compound **5 a** at the end of 24 h incubation period. A similar inhibition trend was observed for the corresponding regioisomer **5 b** (R=3‐OMe, 4‐OH) which showed ~15 %, 33 % and 41 % inhibition of Aβ42 aggregation at 1, 5 and 25 μM respectively (Panel B, Figure 3). Incorporating a 3,4‐dimethoxyphenyl moiety in compound **5 c** (R=3,4‐diOMe) was not favorable and led to a complete loss of inhibition activity (Panel C, Figure 3) as seen previously for the benzofuran‐2‐carboxamide compound **4 c**. Evaluation of the 4‐OMe derivative **5 d** in the Aβ42 aggregation kinetics assay shows that this compound was able to promote Aβ42 fibrillogenesis at 25 μM and led to dramatic increases in the ThT fluorescence compared to Aβ42 alone curve (Panel D, Figure 3). There was ~1.57‐fold increase in Aβ42 fibrillogenesis. This compound was a weak promoter when tested at 1 and 5 μM (Panel D, Figure 3). In order to rule out the possibility of fluorescence quenching and potential interference of test compounds in the ThT fluorescence range (440 and 490 nm), we carried out control experiments with test compounds and ThT (Figure S9, Supporting Information).[^28^, ^29^] These studies further demonstrate that the test compounds (**4 a**–**d** and **5 a**–**d**) exhibit low fluorescence intensities and do not interfere in the ThT based fluorescence kinetics assay.

The aggregation kinetic studies of Aβ42 aggregation inhibitors **4 a**, **4 b**, **5 a** and **5 b** shows that the most likely mechanism of inhibition is the ability of these compounds to engage with prefibrillar Aβ42 aggregates to reduce their self‐assembly and fibril load as indicated by the reductions in the ThT fluorescence intensity in the presence of inhibitor compounds.

### Effect of Test Compounds on Preformed Aβ42 Fibrils

In the next step, we studied the effect of compounds **4 a**–**d** and **5 a**–**d** on preformed Aβ42 fibrils to determine their Aβ42 modulating properties (Figure S10, Supporting Information). This experiment was carried out by using preformed Aβ42 which was incubated for 24 h with the test compounds (**4 a**–**d** and **5 a**–**d** at 25 μM), and the ThT fluorescence was measured after 24 h incubation. Interestingly this study shows that *N*‐phenylbenzofuran‐2‐carboxamide **4 a**, **4 b** and **4 c** and *N*‐phenylbenzo[*d*]thiophene‐2‐carboxamides **5 a**, **5 b** and **5 c** were able to exhibit disaggregation activity (16–66 % disaggregation activity, Figure S10, Supporting Information). In contrast, compounds **4 d** and **5 d** exhibited significant increases in ThT fluorescence (Figure S10, Supporting Information). For example, compound **4 d** led to ~2‐fold increase in the ThT fluorescence compared to preformed Aβ42 fibril control whereas compound **5 d** exhibited ~1.7‐fold increase in ThT fluorescence further confirming their ability to promote Aβ42 fibrillogenesis. Results obtained from these aggregation (Figure 2 and 3) and preformed fibril disaggregation studies (Figure S10, Supporting Information) further demonstrate the ability of *N*‐phenylbenzofuran‐2‐carboxamide and *N*‐phenylbenzo[*d*]thiophene‐2‐carboxamide to modulate Aβ42 aggregation properties.

**Figure 4:**
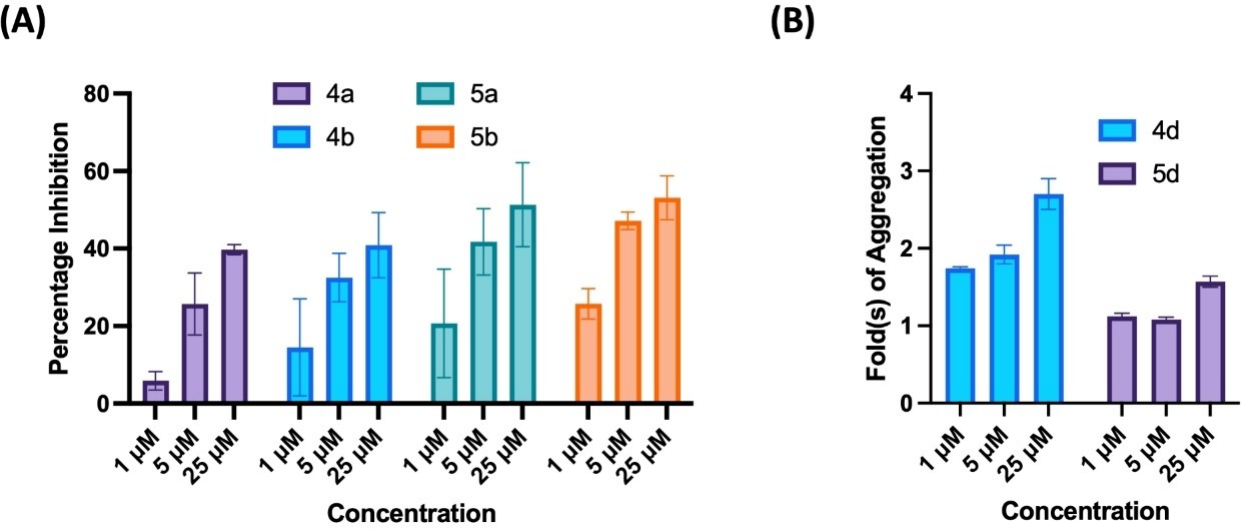
(A) Comparison of percentage inhibition of Aβ42 aggregation inhibitors **4 a**, **4 b**, **5 a**, and **5 b** at 1, 5, 25 μM. (B) Comparison of fold(s) increase in aggregation of Aβ42 by aggregation promoters **4 d** and **5 d** at 1, 5, 25 μM. Results are average±SD based on three independent experiments (n=3).

This study shows that small molecule compounds based on either the *N*‐phenylbenzofuran‐2‐carboxamide or *N*‐phenylbenzo[*d*]thiophene‐2‐carboxamides, have the unique ability to modulate Aβ42 aggregation kinetics and can either prevent or promote their fibrillogenesis depending on the type and position of substituents at the phenyl ring. The presence of a methoxyphenol moiety (compounds **4 a**, **4 b**, **5 a** and **5 b**) provides inhibition and a 4‐methoxyphenyl moiety (**4 d** and **5 d**) causes promotion in Aβ42 fibrillogenesis. The results are further summarized in Figure 4.

### Congo Red Binding Studies

In order to confirm the inhibition/promotion activity of *N*‐phenylbenzofuran‐2‐carboxamide or *N*‐phenylbenzo[*d*]thiophene‐2‐carboxamide derivatives against Aβ42 aggregation observed in the ThT assay, we carried out Congo red (CR) dye binding assay.^[30]^ This assay is used to confirm the formation of β‐sheet structures and to screen compounds that are able to modulate amyloid fibrillogenesis. In this UV based assay, the dye CR, binds to β‐sheet structures present in amyloid proteins which leads to a red shift with the absorbance maxima of CR moving from 490 nm up to 540 nm confirming the presence of β‐sheet structures.[^30^, ^31^, ^32^] Compounds which are able to inhibit the formation of amyloid aggregation are able to prevent the red shift and reduce the absorbance of CR bound to amyloid indicating their ability to inhibit amyloid aggregation.^[29]^ Similarly, compounds that are able to promote Aβ42 fibrillogenesis would promote red shift and enhance CR absorbance. In this regard, we investigated the effect of compounds **4 b**, **4 d**, **5 b** and **5 d** that exhibited superior inhibition or promotion of Aβ42 aggregation in the ThT assay by evaluating their ability to modulate CR binding to Aβ42 aggregates (Figure S11, Supporting Information). After 24 h incubation of Aβ42 (20 μM) with CR dye, there was a significant shift in the maximum absorbance of CR along with a red shift from 490 nm–520 nm and a significant increase in the CR absorbance which further confirms the formation of Aβ42 fibrils (Figure S11, Supporting Information).^[32]^ In contrast, in the presence of 25 μM of either **4 b** or **5 b**, there was a decline in CR absorbance along with an absorbance maxima at 500 nm which supports their ability to reduce Aβ42 aggregation. As observed in the ThT assay, promoter compounds **4 d** and **5 d** were able to demonstrate red shift by moving the CR absorbance maxima to 510 nm and were able to increase CR absorbance demonstrating their ability to promote Aβ42 fibrillogenesis (Figure S11, Supporting Information). This study further confirms the results obtained from the ThT assay.

### Transmission Electron Microscopy (TEM) Studies

**Figure 5:**
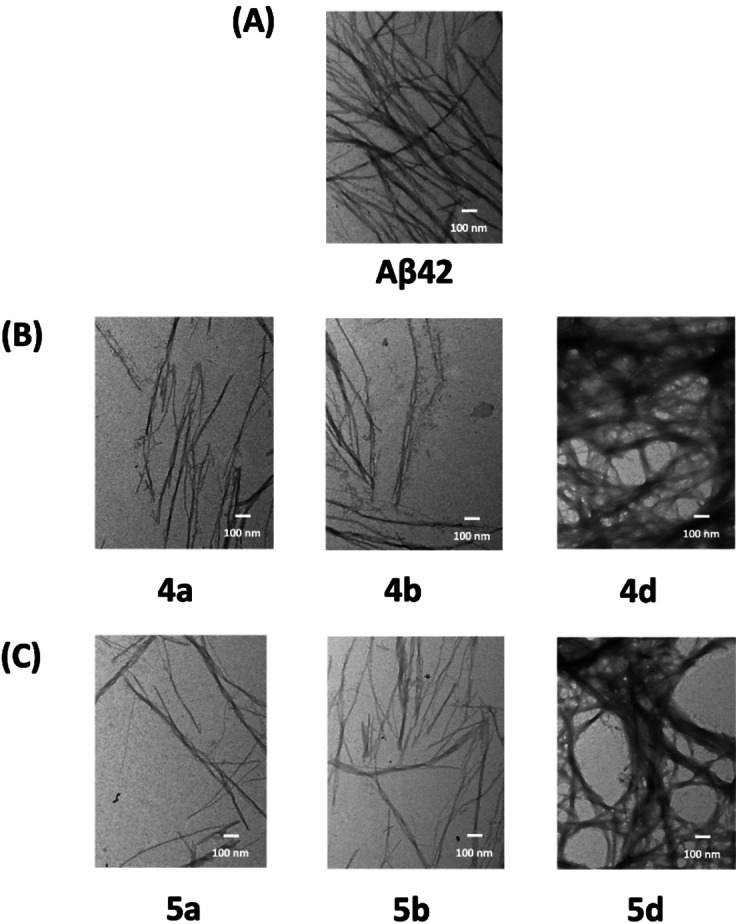
TEM images of Aβ42 (10 μM) in the presence and absence of test compounds. (A) Aβ42 control. (B) Aβ42 with either **4 a**, **4 b** or **4 d** at 25 μM. (C) Aβ42 with either **5 a**, **5 b** or **5 d** at 25 μM. Image scale – 100 nm.

The TEM study is a validated method to confirm the formation of Aβ42 aggregates and also to determine the effect of test compounds on their ability to modulate the fibrillogenesis by observing the changes in Aβ42 aggregate morphology.^[33]^ This method is also considered as an alternative methodology to confirm the results obtained from the ThT based experiments.^[33]^ The effect of *N*‐phenylbenzofuran‐2‐carboxamide or *N*‐phenylbenzo[*d*]thiophene‐2‐carboxamide derivatives **4 a**, **4 b**, **4 d**, **5 a**, **5 b** and **5 d**, on Aβ42 aggregation modulation was further evaluated by carrying out TEM studies. The morphology of Aβ42 aggregates was determined in the presence of 25 μM of test compounds, after incubating them with Aβ42 for a period of 24 h at 37 °C.^[34]^ In the absence of test compounds, a typical morphology of Aβ aggregates was observed which contains densely packed bundles of mature fibrils (Panel A, Figure 5). When Aβ42 was co‐incubated with aggregation inhibitors **4 a**, **4 b**, **5 a** and **5 b** at 25 μM there was a significant reduction in the number of aggregates (Panels B and C, Figure 5) as compared to Aβ42 control (Panel A, Figure 5) which clearly demonstrates the ability of these compounds to prevent Aβ42 aggregation. When Aβ42 fibrillogenesis promoters **4 d** and **5 d** (25 μM each) were co‐incubated for 24 h at 37 °C with Aβ42, more elongated, thread‐like fibrils were observed (Panels B and C, Figure 5). These fibrils are much denser and thicker than those observed for Aβ42 control, which further demonstrates their unique ability to promote Aβ42 fibrillogenesis, and confirms the results obtained from the ThT‐based aggregation kinetics assay. These TEM images provide further evidence on the ability of these *N*‐phenylbenzofuran‐2‐carboxamide or *N*‐phenylbenzo[*d*]thiophene‐2‐carboxamide derivatives to modulate Aβ42 aggregation pathway.

### 8‐Anilino‐1‐Naphthalenesulfonic Acid (ANS) Dye Binding Assay

**Figure 6:**
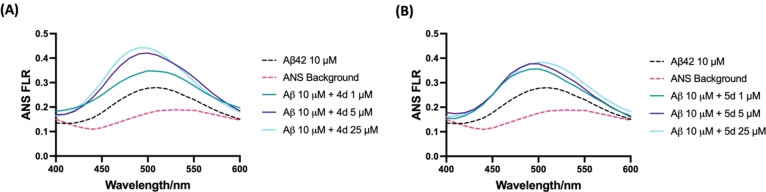
ANS binding assay of **4 d** and **5 d** at 1, 5, 25 μM with Aβ42 (10 μM) after 24 h incubation. (A) ANS spectra of **4 d**. (B) ANS spectra of **5 d**. The ANS FLR was taken at 380 nm excitation and 400–600 nm emission with 10 nm increments. Results are average of three independent experiments (n=3).

Since compounds **4 d** and **5 d** were able to promote Aβ42 fibrillogenesis, we wanted to understand the mechanisms to see if these compounds have the ability to perturb the conformation of Aβ42 aggregates.[^24^, ^35^] In the absence of Aβ42, the ANS dye exhibited maximum emission at 520 nm wavelength (Figure 6). However, in the presence of Aβ42, a blue shift was observed with the maximum emission moving from 520 nm–490 nm along with a significant increase in ANS fluorescence (FLR) intensity which indicates the formation of Aβ42 aggregates exposing ANS binding sites. When Aβ42 was treated with compounds **4 d** and **5 d**, the ANS fluorescence intensity increased in a concentration‐dependent manner with the maximum emission observed at 25 μM (Figure 6). With increasing concentrations (**4 a** at 1, 5, and 25 μM), the ANS fluorescence intensity increased by 1.2, 1.5 and 1.6‐fold respectively compared to Aβ42 control (Panel A, Figure 6). A similar trend was seen for compound **5 b** with 1.3, 1.5, and 1.4‐fold increases in ANS fluorescence seen at 1, 5, 25 μM respectively (Panel B, Figure 6). These results show that compounds **4 d** and **5 d** binding to Aβ42 aggregates can expose additional ANS binding sites. Taken together, these results indicate that benzofuran and benzo[*b*]thiophene carboxamide derivatives **4 d** and **5 d** can perturb the conformation of Aβ42 aggregates and can remodel their aggregation pathways to promote rapid aggregation into β‐sheet rich higher order structures. These results are consistent with our previous work.^[24]^

### Antioxidant Activity of Compounds 4 a, 4 b, 5 a and 5 b

**Figure 7:**
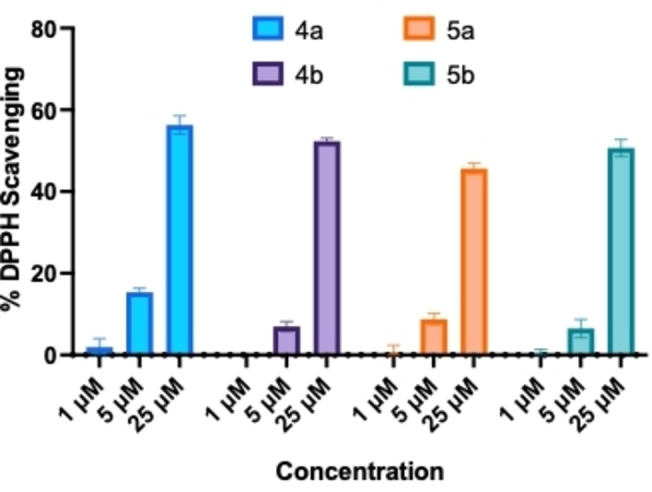
The DPPH free radical scavenging assay for **4 a**, **4 b**, **5 a**, and **5 b** at 1, 5, 25 μM. Results are average of three independent experiments (n=3).

Oxidative stress has long been considered as one of the contributing factors in AD pathogenesis.[^35^, ^36^, ^37^] The aggregation of Aβ fibrils in the brain can result in the release of reactive oxygen species (ROS) which can cause further damage and neurotoxicity.^[37]^ Compounds containing phenolic moiety are often recognized for their antioxidant activities as they can neutralize ROS.^[38]^ Compounds **4 a**, **4 b**, **5 a** and **5 b** posses a methoxyphenol antioxidant pharmacophore, and were evaluated to determine their antioxidant activity by measuring their ability to scavenge the free radical 2,2‐diphenyl‐1‐picryhydrazyl (DPPH).^[39]^ At 1 μM, all these four derivatives exhibited very weak ability to scavenge the free radicals (Figure 7). However, when tested at 5 μM, derivatives **4 a**, **4 b**, **5 a a**nd **5 b** were able to scavenge around 6–15 % DPPH, free radicals (Figure 7). These compounds demonstrated much higher activity when the concentration was increased to 25 μM and led to around 56 %, 52 %, 45 %, and 50 % scavenging activity for **4 a**, **4 b**, **5 a** and **5 b** respectively (Figure 7). These studies demonstrate the ability of these *N*‐substituted phenylbenzofuran and benzo[*b*]thiophene carboxamide derivatives to act as antioxidants.

### Aβ42‐Induced Cell Death Assay in Mouse Hippocampal (HT22) Neuronal Cells

**Figure 8:**
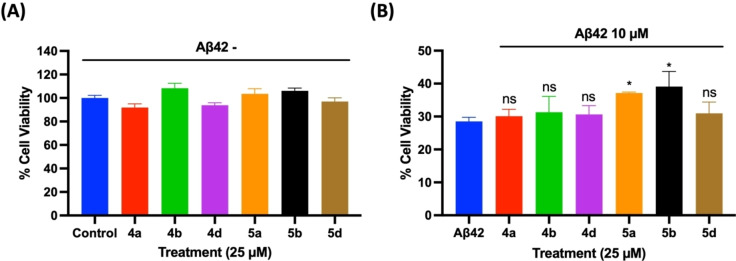
(A) Effects of **4 a**, **4 b**, **4 d**, **5 a**, **5 b**, and **5 d** at 25 μM on mouse hippocampal HT22 neuronal cells in the absence of Aβ42 after 24 h incubation. (B) Neuroprotective effect of derivatives **4 a**, **4 b**, **4 c**, **5 a**, **5 b**, and **5 c** at 25 μM in the presence of Aβ42 (10 μM) after 24 h incubation with HT22 neuronal cells. The cell viability was determined by MTT assay. The results are average of three independent experiments (n=4). ns=not significant, *p<0.01 compared to Aβ42 control (one‐way ANOVA followed by Bonferroni post hoc analysis).

The effect of Aβ42 inhibitors (**4 a**, **4 b**, **5 a** and **5 b**) and promoters (**4 d** and **5 d**) identified from the fluorescence and electron microscopy experiments were evaluated to investigate their ability to protect mouse hippocampal HT22 neuronal cells from Aβ42‐induced cytotoxicity.[^24^, ^40^] The test compounds (**4 a**, **4 b**, **4 d**, **5 a**, **5 b** and **5 d**) were incubated with HT22 cells at 25 μM for a 24 h period at 37 °C to assess their cytotoxicity. These compounds did not show any cytotoxicity to HT22 cells and exhibited cell viability in the range of ~92–108 % compared to untreated cells (Panel A, Figure 8). In the next step, cytotoxicity was induced to HT22 neuronal cells by incubating them with 10 μM of Aβ42 and the cells were incubated for 24 h at 37 °C. Treating the cells with Aβ42 led to significant cell death (28.5 % cell viability Panel B, Figure 8) compared to untreated cells. In the presence of either **4 a**, **4 b** and **4 d** (25 μM each), the cell viability ranged from 30–31 % which shows that these compounds exhibit weak ability to rescue HT22 cells from Aβ42‐induced cytotoxicity. In the presence of benzothiophene derivatives **5 a**, **5 b** and **5 d** (25 μM each), the cell viability ranged from 31–39 % (Panel B, Figure 8). In this regard, both compounds **5 a** and **5 b** exhibited significant protection of HT22 cells from Aβ42‐induced toxicity compared to Aβ42 treated group. It should be noted that although compounds **4 a**, **4 b**, **5 a** and **5 b** exhibited antioxidant activity in the DPPH assay (Figure 7), only compounds **5 a** and **5 b** were able to exhibit significant reduction in Aβ42‐induced toxicity in HT22 neuronal cells. This can be attributed to increased lipophilicity of compounds **5 a** and **5 b** (Log P~3.2, Table S1, Supporting Information) compared to **4 a** and **4 b** (Log P~2.5, Table S1, Supporting Information) which can increase their cell permeability. These molecules have the potential to reduce Aβ42‐induced cytotoxicity by direct binding to Aβ42 thereby reducing the formation of toxic aggregates, and also by indirect mechanisms such as antioxidant activity.[^41^, ^42^]

These cell culture experiments demonstrate that compounds **5 a** and **5 b** are able reduce Aβ42‐induced toxicity in HT22 neuronal cells. This study combined with the aggregation kinetics and TEM study suggests that these compounds are able to bind to prefibrillar aggregates, stabilize their assembly and prevent their further aggregation into more toxic species. Their binding to Aβ42 aggregates can also cause changes in the conformation of Aβ42 aggregates to form less toxic aggregates. In this regard, previous studies have shown that compounds containing planar aromatic rings can increase the surface hydrophobicity of Aβ42 aggregates and can remodel the Aβ42 self‐assembly pathway to form less toxic β‐sheet assemblies.^[43]^

### Fluorescence Imaging Studies in Mouse Hippocampal (HT22) Neuronal Cells

**Figure 9:**
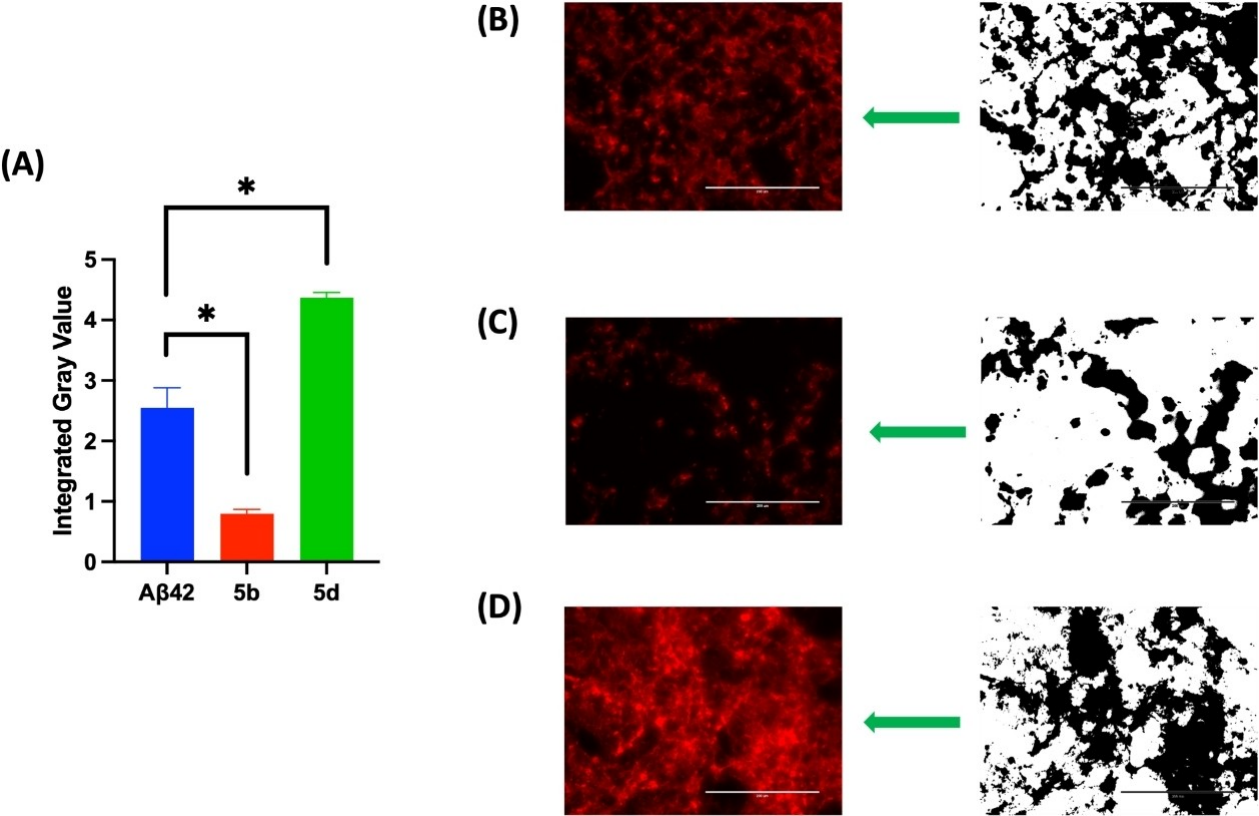
Live cell fluorescence imaging with ProteoStat^®^ dye in the presence and absence of Aβ42 aggregation inhibitor **5 b**, and Aβ42 aggregation promoter **5 d** at 25 μM in HT22 cells. (A) Quantitative analysis of the ProteoStat^®^‐positive stains measured by the integrated gray value (IGV) reflects the amount of Aβ42 aggregates formed in the cellular environment. The results are averages of three independent experiments with three randomized field views at fixed light intensity. *p<0.001 compared to Aβ42 control (one‐way ANOVA followed by Bonferroni post hoc analysis).

Live cell fluorescence imaging was also performed to investigate the effects of compounds **5 b** (Aβ42 aggregation inhibitor) and **5 d** (Aβ42 aggregation promoter) on Aβ42 aggregation in the cellular environment (Figure 9). The fluorescent dye ProteoStat^®^ which is known to bind to Aβ42 aggregates in cell environment was used to stain and quantify the Aβ42 aggregates.^[44]^ The integrated pixel gray value (IGV) of the ProteoStat^®^‐positive stain was calculated as a measure of Aβ42 aggregates formed in the presence and absence of compounds **5 b** and **5 d** (25 μM each), after incubating them with HT22 neuronal cells in the presence of Aβ42 (10 μM).[^24^, ^44^] When the cells were treated with only Aβ42, the IGV was around 2.5 indicating the formation of Aβ42 aggregates (Panels A and B, Figure 9). However, when compound **5 b** (25 μM) was incubated with Aβ42 in HT22 cells, a significantly lower amount of Aβ42 aggregates were seen (IGV~0.9, Panel C, Figure 9). In contrast, in the presence of Aβ42 aggregation promotor compound **5 d**, there was a drastic increase in the amount of Aβ42 aggregates as observed with increased intensity of ProteoStat^®^ stain (IGV 4.0, Panel D, Figure 9) which illustrates its ability to promote and accelerate Aβ42 fibrillogenesis. These cell imaging studies further highlight the Aβ42 aggregation modulatory properties of *N*‐phenylbenzofuran‐2‐carboxamide or *N*‐phenylbenzo[*d*]thiophene‐2‐carboxamide derivatives. Furthermore, calculating the physicochemical properties of these compounds using the web tool SwissADME suggests that this series of compounds have the ability to get into brain with log P values ranging from 2.50–3.62 and exhibit drug‐like properties (Table S1, Supporting Information).^[45]^

### Molecular Docking Studies of 5 b and 5 d in Aβ42 Fibril Assembly

**Figure 10:**
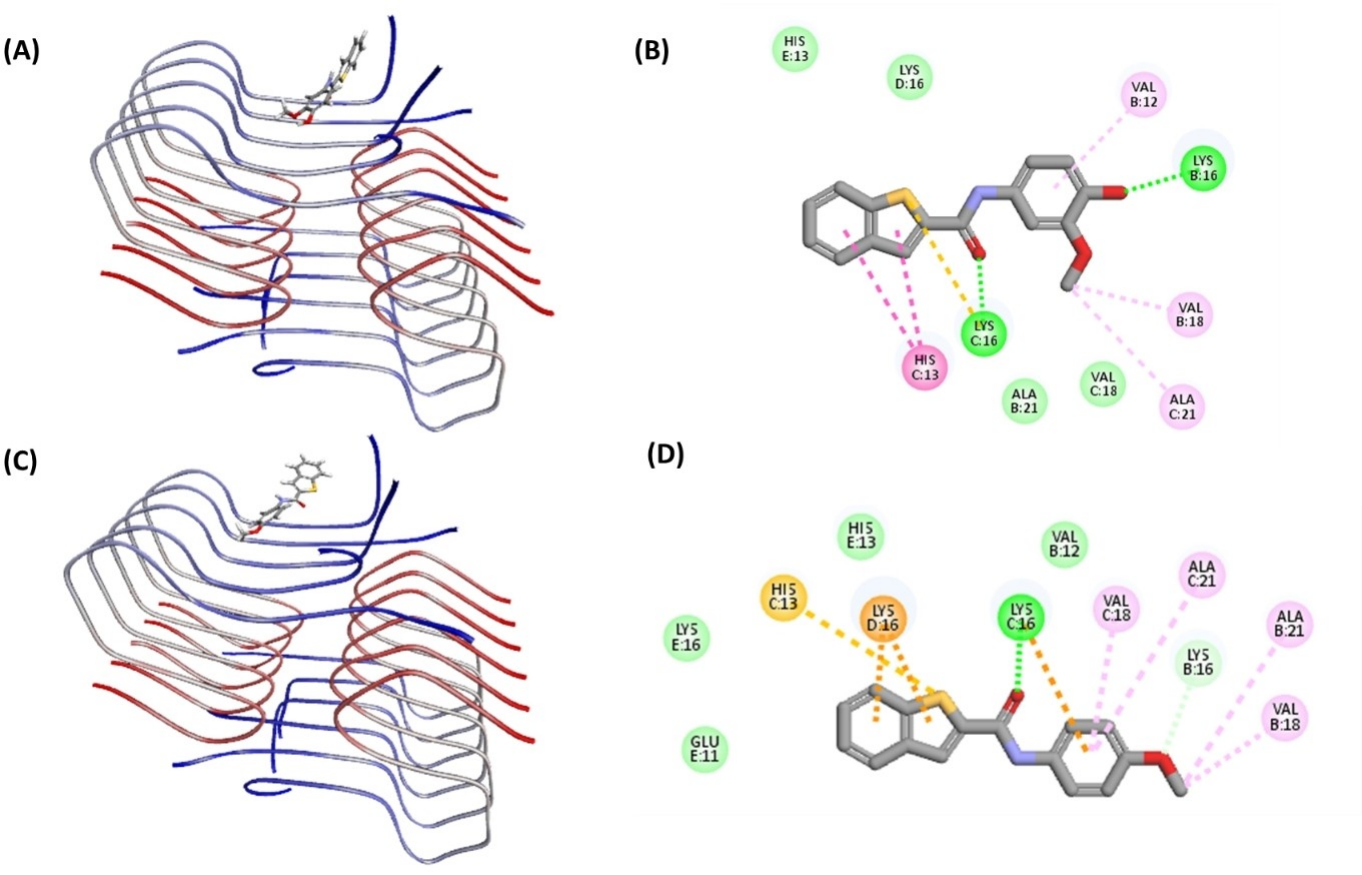
Molecular docking studies of **5 b** and **5 d** in the Aβ42 fibril model (PDB id: 5KK3). (A) Top docking pose of **5 b**. (B) 2D interaction map of the top docking pose of **5 b**. (C) Top docking pose of **5 d**. (D) 2D interaction map of the top docking pose of **5 d**. The hydrogen atoms are removed to enhance clarity. The Aβ42 chains are color coded with blue indicating N‐terminus and red indicating C‐terminus. For the ligand, atoms are color coded with carbon in dark grey, sulfur in yellow, oxygen in red and nitrogen in blue. The interactions are color coded with electrostatic interactions in yellow, hydrophobic interactions in pink, and hydrogen bonding interactions in green.

Molecular docking studies were conducted to investigate the binding interactions of the Aβ42 aggregation inhibitor compound **5 b** and the Aβ42 aggregation promoter **5 d** using the structure of Aβ42 fibril.[^24^, ^39^] The Aβ42 fibril model was prepared from the solved 3D structure of Aβ42 fibril (PDB id: 5KK3).^[14]^ In the top docking pose, compound **5 b** exhibits a planar conformation and was oriented in the N‐terminal region consisting of His13, Lys16, Val18 and Ala21 (CDOCKER energy −13.82 kcal/mol and CDOCKER interaction energy −26.11 kcal/mol, Panel A, Figure 10). Compound **5 b** exhibits a linear conformation such that the bicyclic benzo[*b*]thiophene ring was able to intercalate between the fibril amino acids whereas the methoxyphenol ring was oriented perpendicular to axis of the bicyclic benzo[*b*]thiophene ring. Further analysis shows that the thiophene ring (benzo[*b*]thiophene) underwent cation–π interaction with the positively charged: C: Lys16 side chain (distance~3.5 Å). Other polar interactions (hydrogen bonding) were seen between the amide carbonyl of **5 b** and C: Lys16 (distance<2.0 Å), and with phenolic group of **5 b** and B: Lys16 (distance<2.0 Å, Panel B, Figure 10). These favorable polar intermolecular interactions anchored and oriented the ligand perpendicular to the long axis of the fibril. Other hydrophobic interactions were also observed where the benzo[*b*]thiophene ring underwent pi‐alkyl interactions with C: His13 (distance~5.0 Å), whereas the phenolic ring underwent pi‐alkyl interaction with B: Val12 (distance<5.0 Å). The 3‐OMe substituent also underwent van der Waal's interactions with B: Val18 (distance<4.0 Å) and C: Ala21 (distance<5.0 Å, Panel B, Figure 10). Interestingly, the aromatic benzo[*b*]thiophene ring also underwent T‐shaped π–π interaction with the aromatic ring of C: His13 (distance<5.0 Å) which has a stabilizing effect. The interactions of compound **5 b** with Aβ42 was dominated by polar interactions which is most likely responsible for its ability to inhibit Aβ42 aggregation. In contrast, the top docking pose of aggregation promoter **5 d** shows a different orientation (Panel C, Figure 10) with CDOCKER energy −12.72 kcal/mol and CDOCKER interaction energy −25.93 kcal/mol. Compound **5 d** exhibits a linear and planar conformation. Interestingly, unlike the Aβ42 inhibitor **5 b**, the bicyclic benzo[*b*]thiophene ring of **5 d** was not intercalating with fibril amino acids. Instead, both the benzo[*b*]thiophene and the 4‐methoxyphenyl rings were interacting with fibril amino acids in a face‐to‐face fashion (Panel C, Figure 10). The 4‐methoxylphenyl substituent was oriented in a hydrophobic groove made up of two chains of Aβ42 (B: Val18, B: Ala21, C: Val18, C: Ala21, Panel C, Figure 10) and underwent hydrophobic interactions with C: Val18 (distance~5.0 Å), C: Ala21 (distance~5.0 Å), B: Ala21 (distance<5.0 Å), and B: Val18 (distance<5.0 Å) (Panel D, Figure 10). Furthermore, the aromatic rings of **5 d** including the bicyclic benzo[*b*]thiophene ring and the 4‐methoxyphenyl aromatic rings underwent cation–π interactions with C: Lys16 and D: Lys16 respectively (distance<4.0 Å). Similarly, few hydrogen bonding interactions were also found to anchor the ligand to Aβ42 fibrils, including the one between the amide carbonyl oxygen and C: Lys16 (distance<2.0 Å), and between the 4‐methoxy group and C: Lys16 (distance<2.0 Å) (Panel D, Figure 10). These modeling studies suggest that substitutions in the phenyl ring plays a key role in orienting the bicyclic benzo[*b*]thiophene ring, which seems to modulate their ability to either prevent or promote Aβ42 aggregation.

### Molecular Docking Studies of ThT together with Ligands in the Aβ42 Fibril Assembly

**Figure 11:**
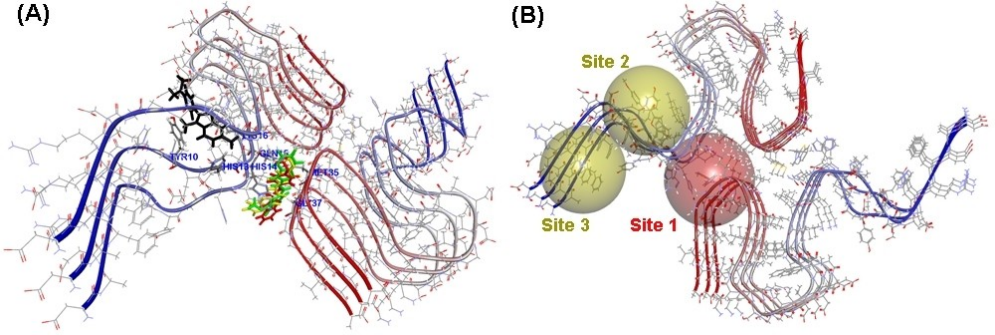
Molecular docking studies of ThT, **4 d**, **5 b** and **5 d** in the full‐length Aβ42 fibril model (PDB id: 5OQV). (A) Top docking poses of ThT (black), **4 d** (yellow), **5 b** (green) and **5 d** (red) in the Aβ42 fibril model. (B) Yellow and red spheres represent binding site locations in the Aβ42 fibril model. Site 1 represents the ligand binding site whereas Site 2 and 3 represent ThT binding sites. The hydrogen atoms are removed to enhance clarity. The Aβ42 chains are color coded with blue indicating N‐terminus and red indicating C‐terminus.

Computational studies were carried out to understand the binding interactions of Aβ42 inhibitor compounds **4 d**, **5 b** and **5 d** in the Aβ42 fibril assembly in the presence of the fluorescent dye ThT to determine the potential binding sites of ThT and the small molecule ligands. The NMR solution structure of the full‐length Aβ42 fibril was used for this study, as these structures represent the conformation of Aβ42 in solution.[^46^, ^47^] The molecular docking studies show that ThT binds on the surface of Aβ42 fibrils as reported previously (Figure 11A).[^30^, ^48^] It was binding in the N‐terminal region, and underwent interactions with Tyr10, His13 and Lys16 whereas the inhibitor compounds **4 d**, **5 b** and **5 d** bind in a narrow channel formed between the C‐ and N‐terminal as shown in Figure 11A. They were in contact with His14, Gln15, Met35 and Gly37. It should be noted that ThT is known to bind on the surface of amyloid fibrils and not at the inner core. Furthermore, ThT is known to bind to multiple sites in amyloid aggregatess.^[48]^ Figure 11B shows two potential sites for ThT binding identified by docking studies. In this regard, our computational study shows that compound **4 b**, **4 d** and **5 d** are able to bind to distinct sites compared to ThT. It is plausible that during the aggregation process, the ligand binding site locations can vary depending on the i) type of aggregates present (eg: oligomers vs fibrils) and ii) the size of aggregates (lower order vs higher order β‐sheet structures).

## Conclusions

This study investigated the SAR of *N*‐phenylbenzofuran‐2‐carboxamide and *N*‐phenylbenzo[*d*]thiophene‐2‐carboxamide derivatives where the phenyl ring was modified structurally by regiosiomeric placement of methoxyphenol, 3,4‐dimethoxyphenyl and 4‐methoxyphenyl substituents. Their evaluation by fluorescence and biophysical experiments demonstrate that the presence of either a 3‐hydroxy‐4‐methoxyphenyl or 4‐hydroxy‐3‐methoxyphenyl ring was a requirement to inhibit Aβ42 aggregation (compounds **4 a**, **4 b**, **5 a** and **5 b**, 41–54 % inhibition at 25 μM). In contrast, the incorporation of a 4‐methoxyphenyl substituent led to acceleration in Aβ42 aggregation (compounds **4 d** and **5 d**), with compound **4 d** exhibiting 2.7–fold increase in aggregation compared to Aβ42 control. These results demonstrate the unique ability of these *N*‐phenylbenzofuran‐2‐carboxamide or *N*‐phenylbenzo[*d*]thiophene‐2‐carboxamide class of compounds as modulators of Aβ42 aggregation. Furthermore, compounds **4 a**, **4 b**, **5 a** and **5 b** possessing the methoxyphenol pharmacophore also exhibited antioxidant activity (45–56 % DPPH scavenging activity at 25 μM). In the cell culture assay compounds **4 a**, **4 b**, **4 d**, **5 a**, **5 b** and **5 d** were nontoxic to mouse hippocampal neuronal HT22 cells at 25 μM. Furthermore, the benzo[*d*]thiophene‐2‐carboxamide compounds **5 a** and **5 b** were able to protect the mouse hippocampal neuronal HT22 cells from Aβ42‐mediated cytotoxicity. Molecular docking studies show that these class of compounds are able to interact in the N‐terminal region of Aβ42 fibrils consisting of His13, Lys16, Val18 and Ala21, and that the planar bicyclic benzofuran or benzo[*d*]thiophene rings can either intercalate in this region, or can undergo face‐to‐face interaction which determines their ability to act as either Aβ42 aggregation inhibitors or promoters. Modeling studies also suggest that the phenyl ring and its substituents play a role in modulating the conformations of these class of compounds while interacting with Aβ42.

Our studies demonstrate that small molecules based on either a *N*‐phenylbenzofuran‐2‐carboxamide or *N*‐phenylbenzo[*d*]thiophene‐2‐carboxamide templates can be tweaked chemically to synthesize novel derivatives which are capable of binding to Aβ42 prefibrillar or fibrillar aggregates and reduce Aβ42 induced cytotoxicity. These molecules have a wide range of applications including i) as chemical/pharmacological tools to study the mechanisms of Aβ42 aggregation; ii) to design novel AD diagnostics (eg: PET imaging agents) and iii) to develop novel small molecule based therapies to treat Alzheimer's disease.

## Experimental Section

### General

The chemicals and reagents used for synthesis were purchased from MilliporeSigma Ltd, Oakville Canada, and AA Blocks, San Diego USA. They were at >95 % pure and used without further purification. The reactions were monitored through thin layer chromatography (TLC) using Merck silica gel 60, F254 with short and long wavelength UV (254 nm and 365 nm respectively). Upon completion, the compounds were purified by column chromatography using Merck silica gel 230–400 mech. The melting points were measured using the digital melting point apparatus, REACH Devices, Boulder USA. The ^1^H NMR (300 MHz) and ^13^C (75 MHz) spectra were obtained on a Brucker Avance spectrometer (Department of Chemistry, University of Waterloo). Either CDCl_3_ or DMSO‐d_6_ were used as the solvents. Coupling constants (*J* values) were recorded in Hertz (Hz). Abbreviations used to represent ^1^H NMR signals were s – singlet, d – doublet, t – triplet, m – multiplet, br s – broad singlet. The mass and purity of compounds were tested on an Agilent 1260 Infinity liquid chromatography module equipped with 6130 quadrupole mass spectrometry using a ZORBAX Eclipse AAA, 4.6×75 mm, 3.5 μm column. A mixture of water and acetonitrile 1 : 1 v/v with 0.1 % formic acid (1.0 mL/min flow rate), was used as the solvent system to assess compound purity and mass. The exact molecular weights of new compounds were determined (High‐Resolution Mass Spectrometry, HRMS) using the Thermo Scientific Q‐Exactive Orbitrap mass spectrometer (positive mode, ESI), Department of Chemistry, University of Waterloo. The reagents used for biological assays were purchased from various vendors. The dye ThT was purchased from MedChemExpress, New Jersey USA, glycine was purchased from Fisher Scientific, Ottawa Canada, ANS was purchased from Cayman Chemical, Michigan USA, Hoechst 33342 was purchased from MilliporeSigma Ltd, Oakville Canada. The ProteoStat assay kit was purchased from Cedarlane, Burlington Canada. Dulbecco's modified eagle medium and nutrient mixture F‐12 (DMEM/F12), fetal bovine serum (FBS), trypsin, penicillin/streptomycin were purchased from Fisher Scientific, Ottawa Canada, The ultra‐pure water (UPW) was purchased from Cayman Chemical, Michigan USA. The Aβ42 1,1,3,3,3‐hexafluoroisopropanol >95 % purity, was purchased from Anaspec (catalog no: AS‐64129‐05), Fremont USA and rPeptide, Georgia USA (catalog no: A‐1163‐2). Compounds **4 c**, **4 d** and **5 d** were previously reported.[^48^, ^49^]

### General Procedure to Synthesize Carboxamide Derivatives 4 a–d and 5 a–d

In a 50 mL round bottom flask, 15 mL of tetrahydrofuran was added. This was followed by the addition of benzofuran‐2‐carboxylic acid (**1**, 1 mmol, Scheme 1 **)** or benzo[*b*]thiophene‐2‐carboxylic acid (**2**, 1 mmol, Scheme 1), 1‐ethyl‐3‐(3‐dimethylaminopropyl)carbodiimide (EDC, 1.5 mmol), hydroxybenzotriazole (HOBt, 1.2 mmol) and triethylamine (TEA, 1.2 mmol). The reaction mixture was kept at room temperature while stirring until all the starting materials and reagents were dissolved. Then, the corresponding aniline derivative (**3 a–d**, 1 mmol) was added slowly to the reaction mixture. The reaction was kept at room temperature overnight, and was monitored by TLC. Upon completion, the solvent was evaporated under reduced pressure. The resulting crude product was extracted with ethyl acetate (15 mL×3) and saturated brine solution (15 mL). The organic layers were collected and dried over with Na_2_SO_4_ and then the salts were filtered. The organic solvent was then evaporated under reduced pressure to obtain the crude product. Further purification was carried out by column chromatography using either ethyl acetate:*n*‐hexane 1 : 1 v/v or ethyl acetate:*n*‐hexane 7 : 3 v/v as the mobile phase. The pure products were analyzed by LCMS to assess their purity. The analytical data and the spectra of *N*‐(substitutedphenyl)benzofuran‐2‐carboxamide and *N*‐(substitutedphenyl)benzo[*b*]thiophene‐2‐carboxamide derivatives derivatives **4 a–d** and **5 a–d** are given below and in the Supporting Information file:

### *N*‐(3‐Hydroxy‐4‐Methoxyphenyl)benzofuran‐2‐Carboxamide (4 a)

The product was obtained as a yellow solid (Yield=80.9 %).

^1^H NMR (300 MHz, DMSO) δ: 10.25 (s, 1H), 9.08 (s, 1H), 7.81–7.74 (m, 1H), 7.70–7.64 (m, 2H), 7.50–7.42 (m, 1H), 7.36–7.28 (m, 2H), 7.14 (d, *J*=8.7 Hz, 1H), 6.86 (d, *J*=8.7 Hz, 1H), 3.72 (s, 3H). mp: 125–128 °C. ESI‐MS, m/z calcd for C_16_H_13_NO_4_ [M+H]^+^ 284.0923, found 284.0917. Purity: 94.3 % (LCMS).

### *N*‐(4‐Hydroxy‐3‐Methoxyphenyl)benzofuran‐2‐Carboxamide (4 b)

The product was obtained as a yellow solid (Yield=86.5 %).

^1^H NMR (300 MHz, CDCl_3_) δ: 8.25 (s, 1H), 7.73–7.66 (m, 2H), 7.58–7.52 (m, 2H), 7.44 (d, *J*=8.5, 1H), 7.31 (d, *J*=8.0 Hz, 1H), 6.91–6.87 (m, 2H), 5.51 (s, 1H), 3.94 (s, 3H). ^13^C NMR (75 MHz, DMSO) δ 156.62, 154.84, 149.60, 147.65, 143.69, 130.78, 127.69, 127.43, 124.26, 123.29, 115.57, 113.69, 112.34, 110.53, 106.49, 56.01. mp: 185–188 °C. ESI‐MS, m/z calcd for C_16_H_13_NO_4_ [M+H]^+^ 284.0923, found 284.0916. Purity: 95.4 % (LCMS).

### *N*‐(3,4‐Dimethoxyphenyl)benzofuran‐2‐Carboxamide (4 c)^[49]^

The product was obtained as a yellow solid (Yield=79.8 %).

^1^H NMR (300 MHz, CDCl_3_) δ: 8.26 (s, 1H), 7.70 (d, *J*=7.7 Hz, 1H), 7.59–7.52 (m, 3H), 7.50–7.39 (m, 1H), 7.35–7.27 (m, 1H), 7.07 (d, *J*=8.6 Hz, 1H), 6.92–6.82 (m, 1H), 3.93 (s, 3H), 3.88 (s, 3H). mp: 129–132 °C. ESI‐MS, m/z calcd for C_17_H_15_NO_4_ [M+H]^+^ 298.1, found 298.0. Purity: 95.9 % (LCMS).

### *N*‐(4‐Methoxyphenyl)benzofuran‐2‐Carboxamide (4 d)^[49]^

The product was obtained as a yellow solid (Yield=82.4 %).

^1^H NMR (300 MHz, DMSO) δ: 10.40 (s, 1H), 7.83–7.75 (m, 1H), 7.73–7.62 (m, 4H), 7.47 (d, *J*=8.6 Hz, 1H), 7.37–7.29 (m, 1H), 6.95–6.88 (m, 2H), 3.72 (s, 3H). mp: 156–159 °C. ESI‐MS, m/z calcd for C_16_H_13_NO_3_ [M+H]^+^ 268.1, found 268.0. Purity: 98.7 % (LCMS).

### *N*‐(3‐Hydroxy‐4‐Methoxyphenyl)benzo[*b*]thiophene‐2‐Carboxamide (5 a)

The product was obtained as a yellow solid (Yield=72.7 %).

^1^H NMR (300 MHz, DMSO) δ: 10.25 (s, 1H), 9.09 (s, 1H), 8.29–8.26 (m, 1H), 8.06–7.89 (m, 2H), 7.49– .38 (m, 2H), 7.28 (d, *J*=2.5 Hz, 1H), 7.11 (dd, *J*=8.7 Hz, 1H), 6.87 (d, *J*=8.8 Hz, 1H), 3.72 (s, 3H). ^13^C NMR (75 MHz, DMSO) δ 160.27, 146.84, 144.84, 140.93, 140.84, 139.66, 132.54, 126.81, 125.78, 125.77, 125.47, 123.29, 112.81, 111.64, 109.24, 56.31. mp: 170–173 °C. ESI‐MS, m/z calcd for C_16_H_13_NO_3_S [M+H]^+^ 300.0694, found 300.0696. Purity: 96.9 % (LCMS).

### *N*‐(4‐Hydroxy‐3‐Methoxyphenyl)benzo[*b*]thiophene‐2‐Carboxamide (5 b)

The product was obtained as a yellow solid (Yield=72.3 %).

^1^H NMR (300 MHz, CDCl_3_) δ: 7.89–7.82 (m, 2H), 7.73 (s, 1H), 7.62 (d, *J*=2.4 Hz, 1H), 7.49–7.36 (m, 2H), 6.88 (d, *J*=8.5 Hz, 1H), 6.79 (dd, *J*=8.5, 2.4 Hz, 1H), 5.52 (br s, 1H), 3.92 (s, 3H). ^13^C NMR (75 MHz, DMSO) δ 160.21, 147.68, 143.60, 140.96, 140.83, 139.67, 131.04, 126.81, 125.75, 125.63, 125.48, 123.31, 115.62, 113.49, 106.27, 55.99. mp: 182–185 °C. ESI‐MS, m/z calcd for C_16_H_13_NO_3_S [M+H]^+^ 300.0694, found 300.0698. Purity: 97.9 % (LCMS).

### *N*‐(3,4‐Dimethoxyphenyl)benzo[*b*]thiophene‐2‐Carboxamide (5 c)

The product was obtained as a yellow solid (Yield=75.5 %).

^1^H NMR (300 MHz, CDCl_3_) δ: 7.91–7.81 (m, 3H), 7.76 (s, 1H), 7.48 (s, 1H), 7.46–7.37 (m, 2H), 7.01–6.94 (m, 1H), 6.87–6.80 (m, 1H), 3.90 (s, 3H), 3.87 (s, 3H). mp: 181–184 °C. ESI‐MS, m/z calcd for C_17_H_15_NO_3_S [M+H]^+^ 313.1, found 314.0. Purity: 95.9 % (LCMS).

### *N*‐(4‐Methoxyphenyl)benzo[*b*]thiophene‐2‐Carboxamide (5 d)^[50]^

The product was obtained as a yellow solid (Yield=89.2 %).

^1^H NMR (300 MHz, CDCl_3_) δ: 7.91–7.83 (m, 3H), 7.68 (s, 1H), 7.55 (s, 1H), 7.52 (s, 1H), 7.48–7.37 (m, 2H), 6.94–6.91 (m, 1H), 6.90–6.87 (m, 1H), 3.81 (s, 3H). mp: 185–188 °C. ESI‐MS, m/z calcd for C_16_H_13_NO_2_S [M+H]^+^ 283.1, found 284.0. Purity: 97.8 % (LCMS).

### Thioflavin T (ThT) Based Aβ42 Aggregation Kinetics Assay

The ability of synthesized *N*‐substituted phenylbenzofuran and benzo[*b*]thiophene derivatives **4 a–d** and **5 a**–**d** to modulate Aβ42 aggregation was evaluated using the ThT assay.[^26^, ^27^] The ThT working solution was prepared in 50 mM glycine buffer using UPW and the pH was adjusted to 7.4. The assay buffer used was prepared by dissolving sodium dibasic phosphate heptahydrate into UPW with a final concentration of 215 mM (pH 7.4). The Aβ42 (Anaspec, USA) was treated with 1 % ammonium hydroxide to obtain 1 mg/mL stock solution and then further diluted to 25 μM working concentration using the assay buffer. Stock solutions of test compounds were prepared in assay buffer and dimethyl sulfoxide was used as the solubilization agent with a final concentration of less than 2 % in the assay wells to obtain 1, 5, and 25 μM working solutions. The aggregation kinetic assay was conducted in the Costar, black clear‐bottom 384 well plate by adding 22 μL ThT working solution and 8 μL Aβ42 solution (10 μM final concentration per well) in the presence and absence of various test compounds (4 μL). The wells were topped off to 40 μL total volume with assay buffer. The plates were covered with a transparent plate cover and were incubated at 37 °C for 24 h with shaking at 300 cpm between readings for 30 s. The RFUs were taken every 10 min (bottom reading), at an excitation wavelength of 440 nm and emission wavelength of 490 nm using the BioTek Synergy H1 microplate reader. Each sample was measured in triplicate readings and the results were obtained based on three independent experiments. The percentage inhibition or promotion of Aβ42 fibrillogenesis was calculated by comparing the ThT fluorescence intensities (RFU) at 24 h. The data were presented as average percent inhibition based on three independent experiments in triplicate readings (n=3).

### Effect of Test Compounds on Preformed Aβ42 Fibrils

The effect of test compounds **4 a–d** and **5 a**–**d** on preformed Aβ42 fibril was evaluated using the ThT assay as described earlier.[^26^, ^27^] The ThT and Aβ42 (Anaspec, USA) stock solutions were prepared, and Aβ42 (10 μM final concentration per well) was allowed to aggregate over a 24 h time period at 37 °C with shaking at 300 cpm in a Costar, black clear‐bottom 384 well plate. Compound stock solutions were prepared in phosphate buffer pH 7.4 μM after using DMSO to solubilize. The ThT fluorescence intensities (RFUs) were measured at an excitation wavelength of 440 nm and emission wavelength of 490 nm using the BioTek Synergy H1 microplate reader as before. After 24 h, test compounds (25 μM final compound concentration per well) were added to Aβ42 wells and incubated at 37 °C with shaking for another 24 h and the ThT RFUs were measured. The percentage disaggregation or promotion of Aβ42 aggregation was calculated by comparing the ThT fluorescence intensities (RFU) with preformed Aβ42 control. The data were presented as average percent disaggregation or promotion of Aβ42 fibrillogenesis based on two independent experiments in triplicate readings (n=3).

### Congo Red (CR) Binding Studies

The CR assay was carried out as per a previously reported method. A 10 mM stock solution of CR was prepared in DMSO.^[32]^ This stock solution was used to prepare CR solution (125 μM) by serial dilution with PBS (pH 7.4) and was stored away from light. Stock solutions of test compounds **4 b**, **4 d**, **5 b**, **5 d** and reference compounds such as resveratrol (RVT) and orange G (OG), were also prepared in DMSO with subsequent dilution with PBS buffer. The final DMSO concentration per well was below 3 % v/v. The Aβ42 • NH_4_OH (rPeptide, USA), >95 % pure, was treated with a 2 % ammonium hydroxide solution to yield a 1 mg/mL stock solution. The Aβ42 stock solution was vortexed and sonicated for 5 minutes before being further diluted to a 50 μM working solution using the PBS assay buffer. The test compounds are incubated with Aβ42 (8 μl, 20 μM final well concentration) under agitation at 37 °C for 24 h. Afterward, the prepared CR solution (4 μl per well, 12.5 μM) was added to all the wells and incubated for an additional 15 minutes at 37 °C. UV absorbance readings were taken from 450–600 nm using the BioTek Synergy H1 microplate reader. Each samples were kept in triplicates and the results were obtained based on two independent experiments.

### Transmission Electron Microscopy (TEM) Experiments

The TEM studies were carried out according to the ThT aggregation kinetic protocol.^[27]^ After 24 h incubation, the Aβ42 solution (10 μM) with or without various test compounds were loaded (20 μL each) onto the 400 mesh formvar‐coated copper grids (Electron Microscopy Sciences, USA). The grids were left to dry overnight and then washed three times with 20 μL UPW. Then the grids were further air‐dried overnight before staining with the 2 % phosphotungstic acid (PTA) solution. Specifically, 20 μL of 2 % PTA was added and the grids were stained for 1 min before removing the PTA by blotting with filter paper. The imaging studies were carried out using a Philips CM 10 TEM (Dept. of Biology, University of Waterloo) at 60 kV and the micrographs were obtained through a 14‐megapixel AMT camera at 60,000X magnification.

### 8‐Anilino‐1‐Naphthalenesulfonic Acid (ANS) Dye Binding Assay

The dye ANS was used to probe the ability of compounds **4 d** and **5 d** to change of Aβ42 conformation.[^24^, ^34^] The working solution of Aβ42 and derivatives **4 d** and **5 d** at 1, 5, 25 μM, and assay buffer (sodium dibasic phosphate heptahydrate, pH 7.4) were prepared as per the ThT aggregation kinetic assay protocol. The ANS dye was dissolved in UPW and further diluted down to 100 μM working solution. The assay was conducted in the Costar black, clear‐bottom 384 well plate by adding 8 μL Aβ42 working solution (10 μM final concentration per well) in the presence and absence of various test compounds (4 μL). The wells were topped off to 40 μL total volume with assay buffer. The plates were covered with a transparent plate cover and were incubated at 37 °C for 24 h with shaking at 300 cpm between readings for 30 s. At the end of the assay, 4 μL of ANS working solution was added to each wells followed by a 10 min incubation at 37 °C with shaking at 700 cpm. The ANS RFUs were acquired using the Thermo Scientific Varioskan LUX multimode plate reader at 380 nm excitation and 400 nm–600 nm emission with 10 nm increments. The results were presented as average RFUs (±standard deviation, SD) based on triplicate measurements from three independent experiments.

### Free Radical Scavenging DPPH (Antioxidant) Assay

The antioxidant activities of compounds **4 a**, **4 b**, **5 a** and **5 b** were evaluated by the DPPH scavenging assay.^[39]^ The DPPH reagent was prepared by dissolving DPPH in methanol to obtain 0.09 mM stock solution. The test compounds were prepared by serial dilution with methanol to obtain 1, 5, 10, 25 μM stock solutions. The assay was conducted in the transparent 96‐well plate and protected from light exposure. Compound background control was taken by adding 100 μL methanol and 25 μl of compound stock solutions at various testing concentrations. For the DPPH control group, 100 μL DPPH stock solution and 25 μL methanol were added into the wells. For compound screening, similarly, 100 μL DPPH stock solution with 25 μL compound dilution at various concentrations was added. The well plate was covered with aluminum foil and kept shaking at room temperature at 700 cpm for 60 min. After incubation period, the absorbance readings were taken at 517 nm. The percentage DPPH scavenging activity was calculated by the following formula: % DPPH scavenging activity=[(Absorbance reading of DPPH control – Absorbance reading of compound screening)/ Absorbance reading of DPPH control]×100 %. Each sample was measured in triplicate readings and the results were obtained based on three independent experiments.

### Aβ42‐Induced Cell Death Assay in Mouse Hippocampal (HT22) Neuronal Cells

The cell death induced by Aβ42 in mouse hippocampal HT22 neuronal cells was evaluated using the 3‐(4,5‐dimethylthiazol‐2‐yl)‐2,5‐diphenyltetrazolium bromide (MTT) assay as per our previous work.[^24^, ^40^] Aβ42 (Catalog number: A‐1163‐2, rPeptide, Georgia, USA) was dissolved in HFIP to a concentration of 1 mg/mL, then aliquoted into microcentrifuge tubes, and allowed to evaporate under desiccant for 24 h. The aliquots were stored at −20 °C with desiccant until further use. Immediately before use, the Aβ42 film was resuspended in DMSO at a concentration of 5 mM, vortexed for 30 s, and then sonicated for 10 min at room temperature. The 5 mM Aβ monomer solution prepared in DMSO was then diluted to 100 μM in cold neurobasal media and incubated at 4 °C for 24 h to form oligomers for treatments. The dye MTT was purchased from MilliporeSigma, Oakville Canada, and was employed to evaluate the toxicity of Aβ42 post‐treatment with test compounds. The HT22 cells were seeded in 96‐well cell culture plates at a density of 100,000 cells/mL in the complete growth medium (DMEM/F12 supplemented with 10 % FBS and 1 % penicillin/streptomycin) and maintained at 37 °C with 5 % CO_2_ for 24 h to allow for cell adhesion and stabilization. Following the initial incubation period, the cell culture medium was replaced with the supplemented neurobasal medium (Neurobasal media, 1x N‐2 supplement, 1x GlutaMax) for an additional 24 h. Subsequently, cells were treated with Aβ42 (10 μM) along with the respective compounds (**4 a**, **4 b**, **4 d**, **5 a**, **5 b** and **5 d**, at 25 μM), and were returned to the incubator for an additional 24 h. For the post‐treatment incubation, the cell culture medium was replaced with phenol red‐free DMEM containing 0.5 mg/mL MTT, and the cells were incubated for a further 3.5 hours at 37 °C in a 5 % CO_2_. The cells were then solubilized in 100 μL of MTT solubilization buffer composed of 90 % isopropanol, 10 % Triton X‐100, and 0.1 % hydrochloric acid. The absorbance measurements were obtained using a spectrophotometric plate reader at wavelengths of 570 nm and 690 nm, with the background signal at 690 nm being subtracted from the absorbance values at 570 nm for the data analysis. The percent cell viability was calculated based on three independent experiments (n=4).

### Fluorescence Imaging Studies in Mouse Hippocampal (HT22) Neuronal Cells

Fluorescence cell imaging was conducted to visualize Aβ42 aggregates in the presence and absence of compounds **5 b** and **5 d**. The amyloid selective red fluorescent dye ProteoStat^®^ was used to stain Aβ42 aggregates.[^24^, ^44^] The mouse hippocampal neuronal HT22 cell lines were cultured and treated as per cell viability assay protocol. At the end of the 24 h treating period, the cells were washed three times with 100 μL PBS. The counter‐staining solution containing the dye was prepared by adding 0.5 μL ProteoStat^®^ into 100 μL phenol red‐free DMEM for each well. The cells were left for staining for 20 min in the incubator protected from light. After staining, the cells were washed with 100 μL PBS for three times with 5 min incubation in between. At the end, 100 μL phenol red‐free DMEM was added into the wells while imaging. The EVOS^®^ FL Auto imaging system equipped with different light filters was used for imaging. ProteoStat^®^ staining was visualized with the red fluorescent protein (RFP) light filter in the microscope. For quantitative analysis, the light intensity was fixed at level while taking images. All the following adjustments and analysis were conducted in the ImageJ software. First, the RFP channel images were converted into 8‐bit monochrome images. Then the threshold was determined automatically with the Huang algorithm with the resulting binary images used as masks to measure the integrated gray value intensity of pixels in the original image. The results were presented as integrated pixel intensity (±standard deviation, SD) based on triplicate measurements from three independent experiments.

### Molecular Docking Studies in Aβ42 Fibril Model

Molecular docking studies were conducted to understand the binding interactions of compounds **5 b** and **5 d** with Aβ42 fibril model. Discovery Studio software, *Structure‐Based‐Design* (BIOVIA Inc. San Diego, USA) was used for molecular docking.[^24^, ^39^] The Aβ42 fibril model was extracted and prepared from the solved 3D structure of Aβ42 fibril (PDB id: 5KK3)^[14]^ using the *Macromolecule* module. A binding sphere with 20 Å radius was created and defined as the ligand binding site which covers the essential hydrophobic domains that are responsible for its aggregation propensity. Compounds **5 b** and **5 d** were built in 3D using the *Small Molecule* module in the software and were subjected to energy minimization using the *Simulation* module (1000 steps of steepest descent followed by 2000 steps of conjugate gradient) and CHARMm force field. The CDOCKER algorithm was used for docking to give 10 docking poses per ligand. The docking poses were ranked based on the CDOCKER energy and the CDOCKER interaction energy in kcal/mol. The top docking poses obtained for compounds **5 b** and **5 d** were further analyzed by investigating polar and nonpolar contacts with the protein. Docking of ThT and ligands **4 d**, **5 b** and **5 d** in the Aβ42 fibril model (PDB id: 5OQV)^[46]^ was carried out using the LibDock algorithm in Discovery Studio software. The three binding sites (Site 1–3) were prepared by selecting amino acids Val34, Lys16‐Ala21, and Phe4 in the fibril model to create 15 Å radius sphere. The top docking poses were analyzed by studying the binding site locations and interactions.

## Conflict of Interests

All the authors declare no conflict of interest.

1

## Supporting information

As a service to our authors and readers, this journal provides supporting information supplied by the authors. Such materials are peer reviewed and may be re‐organized for online delivery, but are not copy‐edited or typeset. Technical support issues arising from supporting information (other than missing files) should be addressed to the authors.

Supporting Information

## Data Availability

The data that support the findings of this study are available from the corresponding author upon reasonable request. ;
