# Supplementary material for: Benzofuran and Benzo[b]thiophene‐2‐Carboxamide Derivatives as Modulators of Amyloid Beta (Aβ42) Aggregation
Source: ChemMedChem. 2024 Sep 30;19(22):e202400198. doi: 10.1002/cmdc.202400198 (PMC11581421; doi:10.1002/cmdc.202400198)
Supplement: Supplementary file 1 — Supporting Information [file CMDC-19-e202400198-s001.pdf]

ChemMedChem

Supporting Information

Benzofuran and Benzo[*b*]thiophene-2-Carboxamide Derivatives as Modulators of Amyloid Beta (Aβ42) Aggregation

Yusheng Zhao, Kartar Singh, Rahul Chowdary Karuturi, Ahmed A. Hefny, Arash Shakeri, Mike A. Beazely, and Praveen P. N. Rao*

Supporting Information

Benzofuran and Benzo[*b*]thiophene-2-carboxamide Derivatives as Modulators of Amyloid Beta (Aβ42) Aggregation

Yusheng Zhao, [a] Kartar Singh, [a] Rahul Chowdary Karuturi, [a] Ahmed A. Hefny, [a] Arash Shakeri, [a] Mike A. Beazely, [a] Praveen P. N. Rao* [a]

Corresponding author: Praveen P. N. Rao, Tel: +1 519 888 4567 ext: 21317; e-mail: praveen.nekkar@uwaterloo.ca

[a] School of Pharmacy, Health Sciences Campus, University of Waterloo, 200 University Avenue West, Waterloo, Ontario, Canada N2L 3G1

Contents

1. Analytical data for *N*-(3-hydroxy-4-methoxyphenyl)benzofuran-2-carboxamide (**4a**)...... 3

2. Analytical data for *N*-(4-hydroxy-3-methoxyphenyl)benzofuran-2-carboxamide (**4b**) 5

3. Analytical data for *N*-(3,4-dimethoxyphenyl)benzofuran-2-carboxamide (**4c**)................. 7

4. Analytical data for *N*-(4-methoxyphenyl)benzofuran-2-carboxamide (**4d**) 8

5. Analytical data for *N*-(3-hydroxy-4-methoxyphenyl)benzo[b]thiophene-2-carboxamide (**5a**) ……………………………………………………………………………………9

6. Analytical data for *N*-(4-hydroxy-3-methoxyphenyl)benzo[b]thiophene-2-carboxamide (**5b**)……...11

7. Analytical data for *N*-(3,4-dimethoxyphenyl)benzo[*b*]thiophene-2-carboxamide (**5c**).... 13

8. Analytical data for *N*-(4-methoxyphenyl)benzo[*b*]thiophene-2-carboxamide **(5d)** 14

9. ThT-based aggregation kinetics background readings... 15

10 Effect of test compounds on preformed Aβ42 fibrils.. 15

11 Congo red UV assay for compounds **4b**, **4d**, **5b**, **5d** and resveratrol (RVT)……………17

12 Physicochemical properties of compounds **4a**–**d** and **5a**–**d**……………………………………………………………………………………18

1. Analytical data for *N*-(3-hydroxy-4-methoxyphenyl)benzofuran-2-carboxamide (4a)

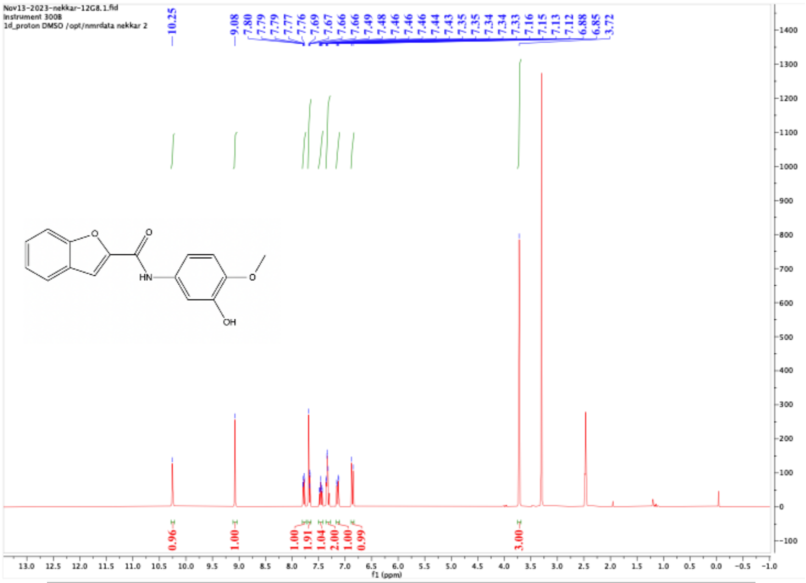

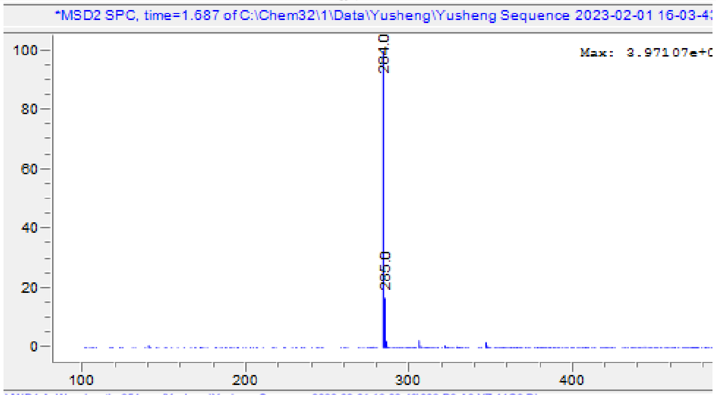

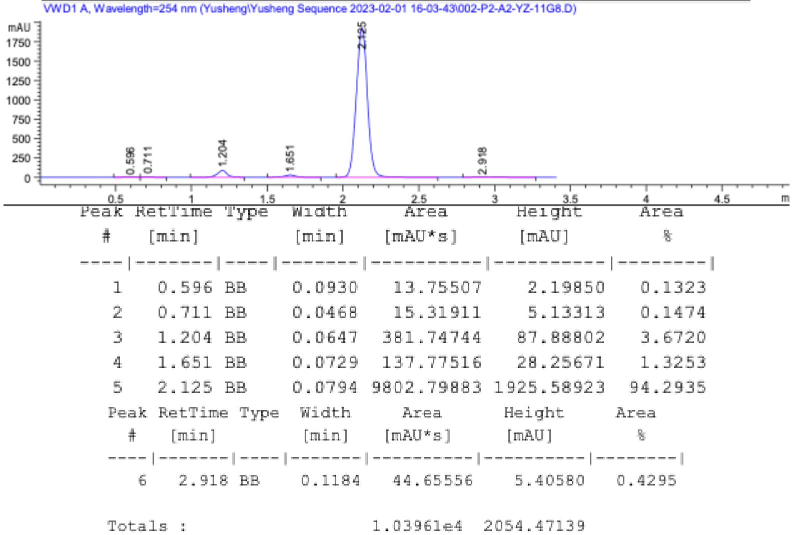

Peak #	RetTime [min]	Type	Width [min]	Area [mAU*s]	Height [mAU]	Area %
1	0.596	BB	0.0930	13.75507	2.19850	0.1323
2	0.711	BB	0.0468	15.31911	5.13313	0.1474
3	1.204	BB	0.0647	381.74744	87.88802	3.6720
4	1.651	BB	0.0729	137.77516	28.25671	1.3253
5	2.125	BB	0.0794	9802.79883	1925.58923	94.2935
6	2.918	BB	0.1184	44.65556	5.40580	0.4295
Totals :				1.03961e4	2054.47139	

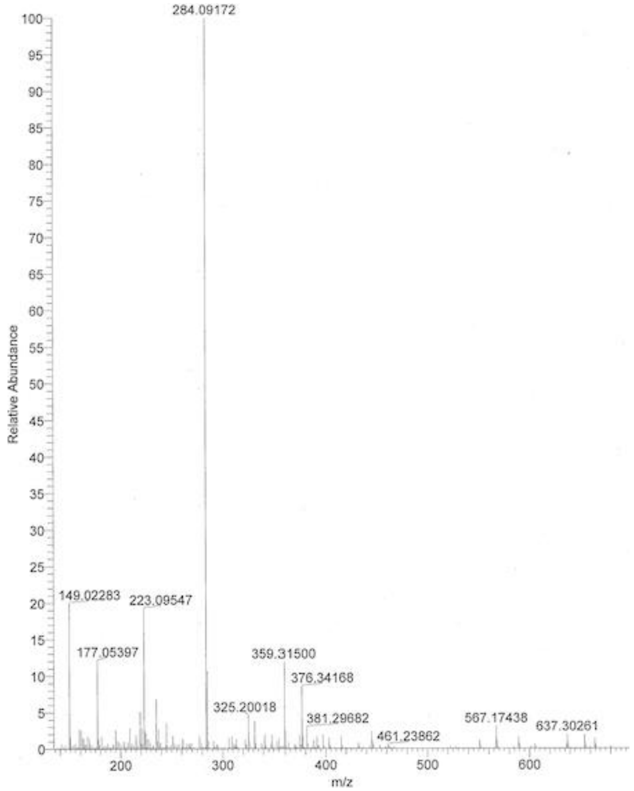
284.09172
149.02283
223.09547
177.05397
359.31500
376.34168
325.20018
381.29682
461.23862
567.17438
637.30261
Relative Abundance
m/z

2. Analytical data for *N*-(4-hydroxy-3-methoxyphenyl)benzofuran-2-carboxamide (4b)

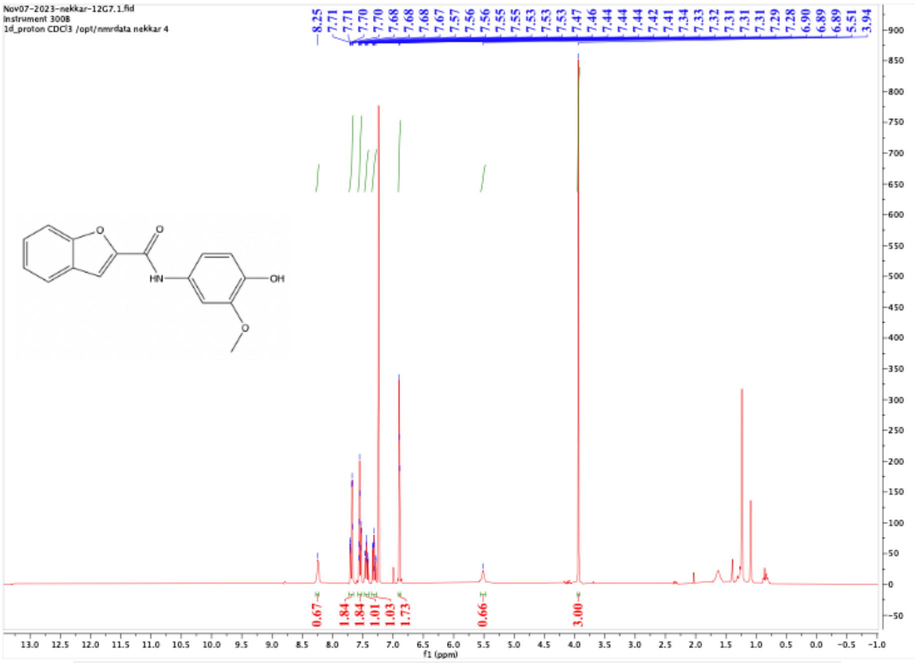

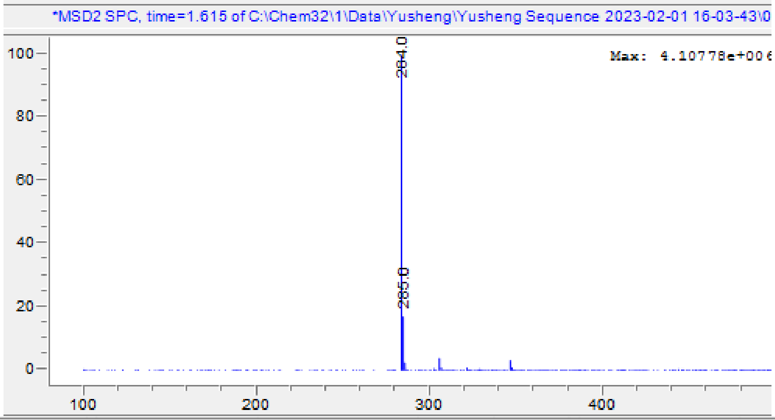

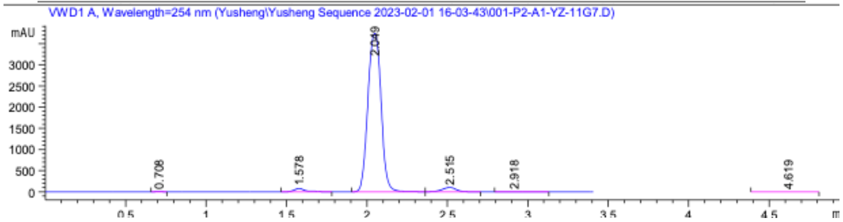

Peak #	RetTime [min]	Type	Width [min]	Area [mAU*s]	Height [mAU]	Area %
1	0.708	BB	0.0430	15.61483	5.87745	0.0702
2	1.578	BB	0.0734	379.87283	77.19826	1.7069
3	2.049	BB	0.0912	2.12289e4	3738.49683	95.3879
4	2.515	BB	0.0900	599.76508	102.93907	2.6949
5	2.918	BB	0.1040	11.98579	1.72714	0.0539
6	4.619	BB	0.1648	19.18929	1.83861	0.0862
Totals :				2.22553e4	3928.07736	

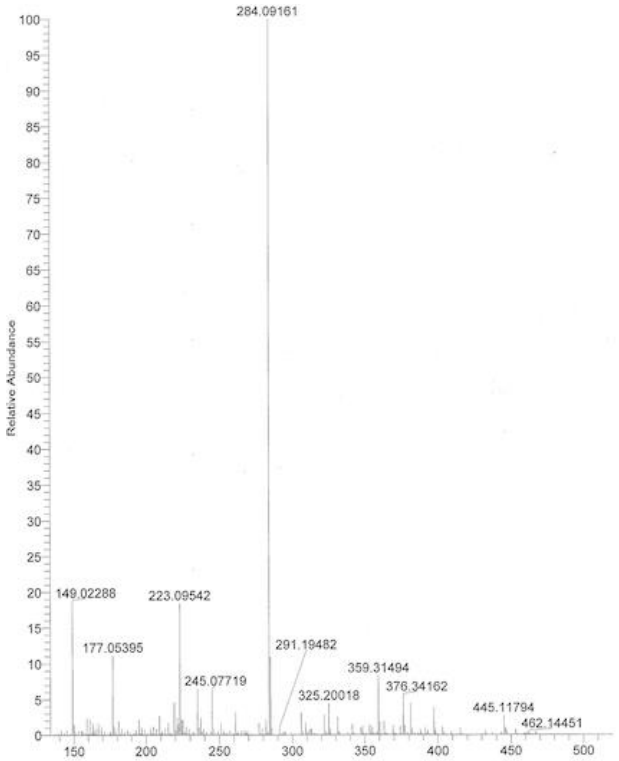

284.09161
149.02288
223.09542
177.05395
291.19482
359.31494
245.07719
376.34162
325.20018
445.11794
462.14451
Relative Abundance
m/z

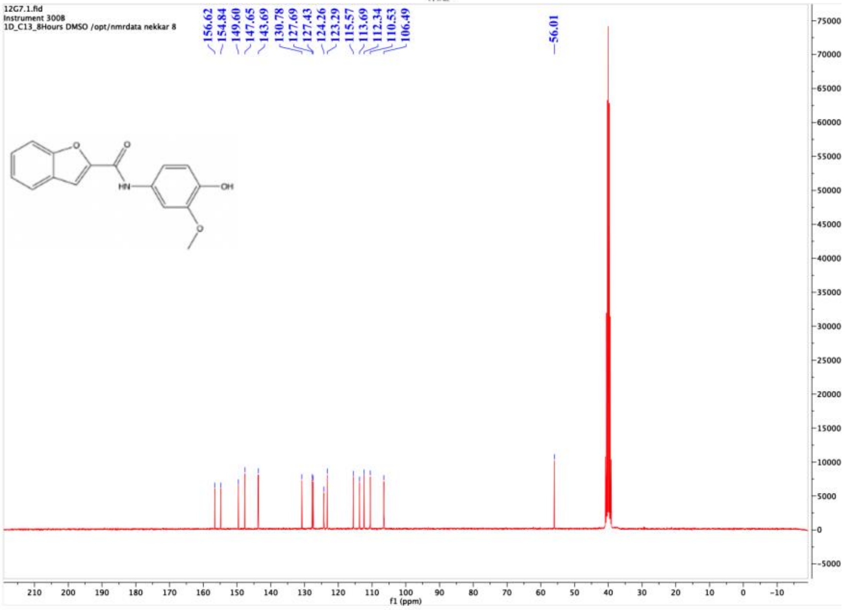

12G7.1.fid
Instrument 3008
1D_C13_8Hours DMSO /opt/nmrdata nekkar 8
156.62
154.84
149.60
147.65
143.69
130.78
127.69
127.43
124.26
123.29
115.57
113.69
112.34
110.53
106.49
56.01
HN
OH
O
f1 (ppm)

3. Analytical data for *N*-(3,4-dimethoxyphenyl)benzofuran-2-carboxamide (4c)

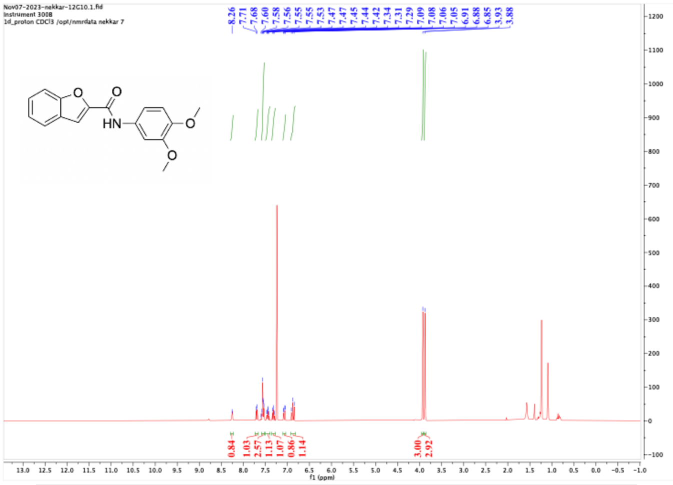

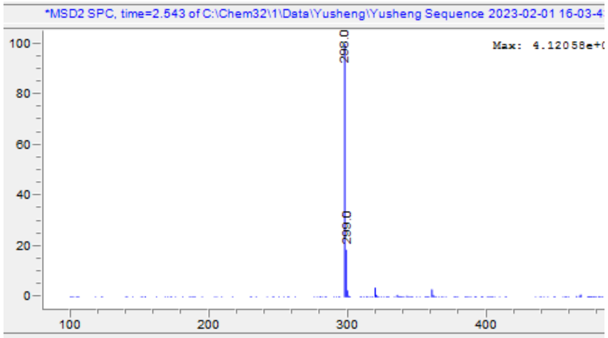

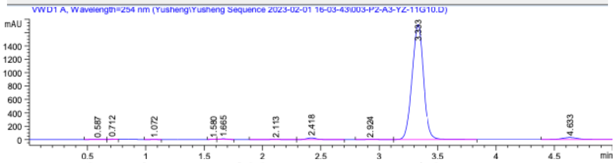

Peak #	RetTime [min]	Type	Width [min]	Area [mAU*s]	Height [mAU]	Area %
1	0.587	BB	0.1088	25.55523	3.98546	0.2094
2	0.712	BB	0.0445	12.27538	4.55153	0.1006
3	1.072	BB	0.0589	7.05302	1.87513	0.0578
4	1.580	BB	0.0416	4.48856	1.76673	0.0368
5	1.665	BB	0.0612	30.60577	8.08403	0.2508
6	2.113	BB	0.1003	18.67484	2.78120	0.1531
7	2.418	BB	0.0846	132.72072	24.34251	1.0877
8	2.924	BB	0.1104	14.17255	1.87020	0.1162
9	3.333	BB	0.1054	1.17001e4	1719.84351	95.8882
10	4.633	BBA	0.1357	256.16074	29.09146	2.0994

Peak #	RetTime [min]	Type	Width [min]	Area [mAU*s]	Height [mAU]	Area %
Totals :				1.22018e4	1798.19176	

4. Analytical data for *N*-(4-methoxyphenyl)benzofuran-2-carboxamide (4d)

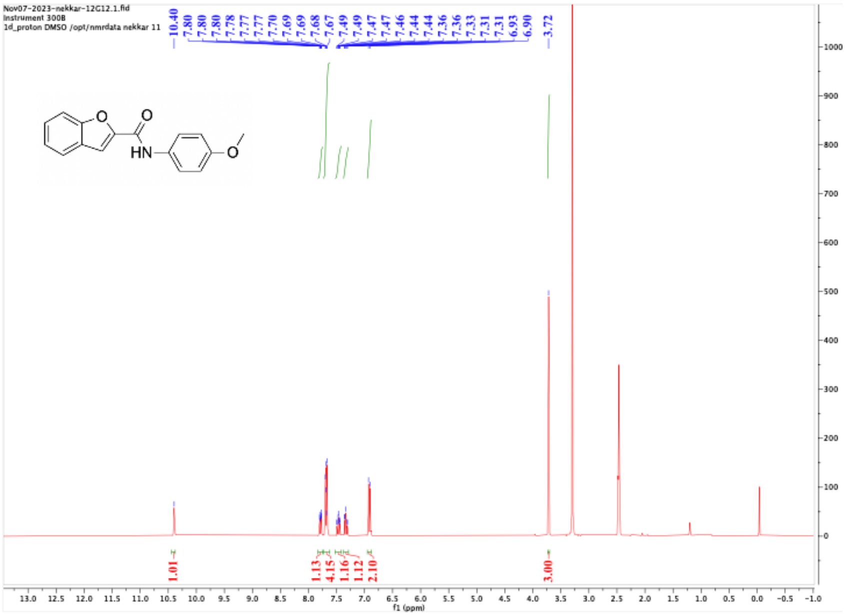

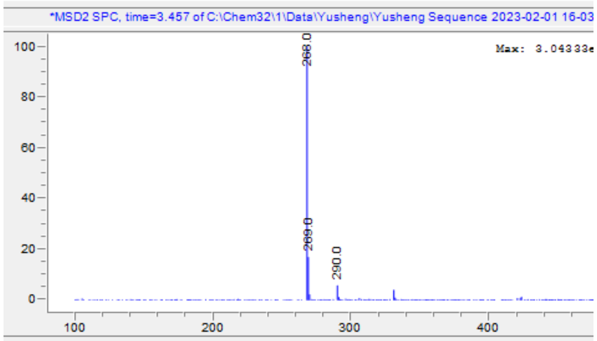

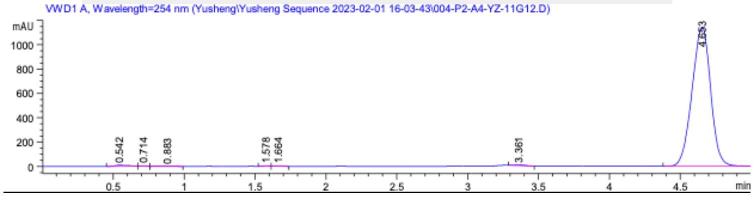

Peak #	RetTime [min]	Type	Width [min]	Area [mAU*s]	Height [mAU]	Area %
1	0.542	BB	0.0863	59.07301	9.15759	0.5616
2	0.714	BB	0.0387	9.52620	4.00770	0.0906
3	0.883	BB	0.0954	15.68039	2.22070	0.1491
4	1.578	BB	0.0453	5.91851	2.13864	0.0563
5	1.664	BB	0.0581	12.74531	3.52930	0.1212
6	3.361	BB	0.0868	30.76379	5.71166	0.2925
7	4.653	BBA	0.1420	1.03850e4	1142.70837	98.7289
Totals :				1.05187e4	1169.47398	

5. Analytical data for *N*-(3-hydroxy-4-methoxyphenyl)benzo[b]thiophene-2-carboxamide (5a)

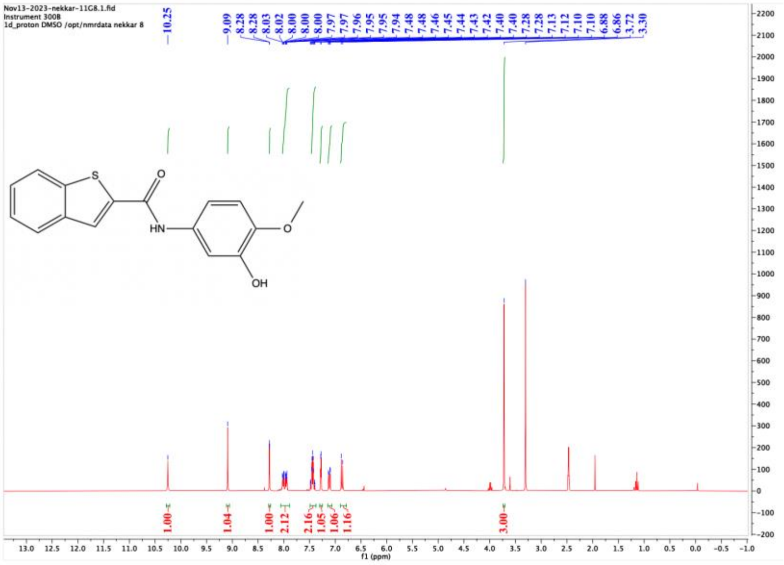

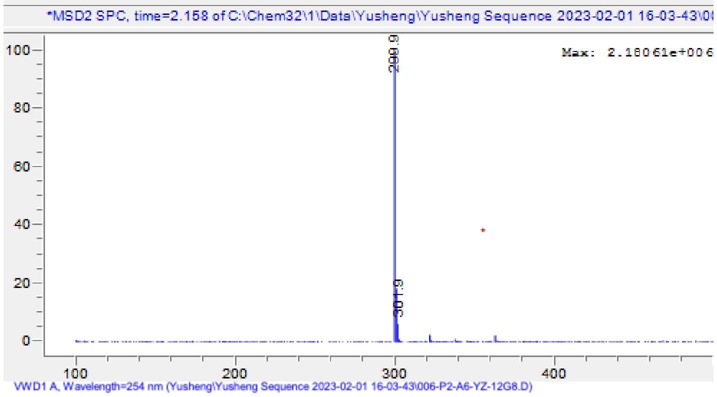

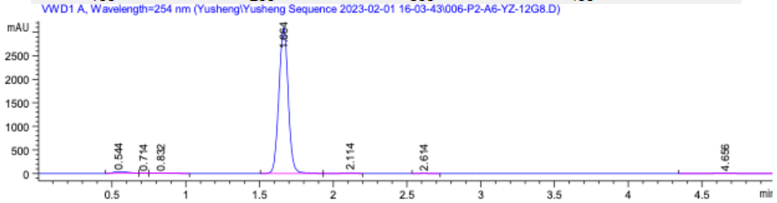

Peak #	RetTime [min]	Type	Width [min]	Area [mAU*s]	Height [mAU]	Area %
1	0.544	BB	0.0924	244.85555	36.88563	1.7449
2	0.714	BB	0.0345	4.53921	2.16488	0.0323
3	0.832	BB	0.1312	54.95166	6.46300	0.3916
4	1.664	BB	0.0684	1.36022e4	3089.33960	96.9346
5	2.114	BB	0.0751	45.44403	9.45236	0.3239
6	2.614	BB	0.0778	15.67872	3.22137	0.1117
7	4.656	BBA	0.1379	64.68129	7.26115	0.4609

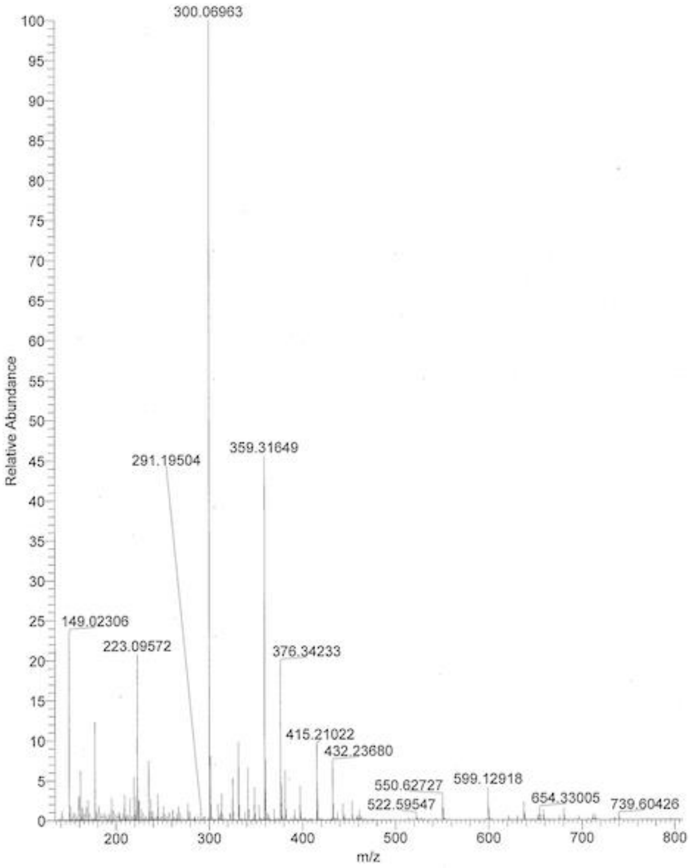
300.06963
359.31649
291.19504
149.02306
223.09572
376.34233
415.21022
432.23680
550.62727
522.59547
599.12918
654.33005
739.60426
Relative Abundance
m/z

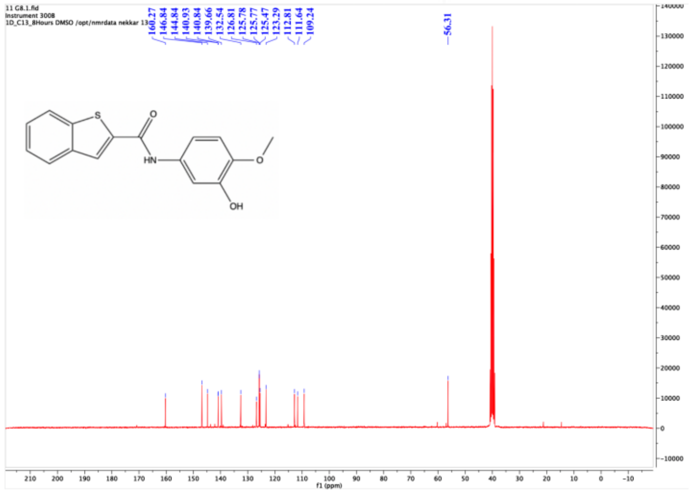
11 G8.1.fid
Instrument 3008
1D_C13_8Hours DMSO /opt/nmrdata nekkar 13
160.27
146.84
144.84
140.93
140.84
139.66
132.54
126.81
125.78
125.77
125.47
123.29
112.81
111.64
109.24
56.31
S
O
HN
O
OH
f1 (ppm)

6. Analytical data for *N*-(4-hydroxy-3-methoxyphenyl)benzo[b]thiophene-2-carboxamide (5b)

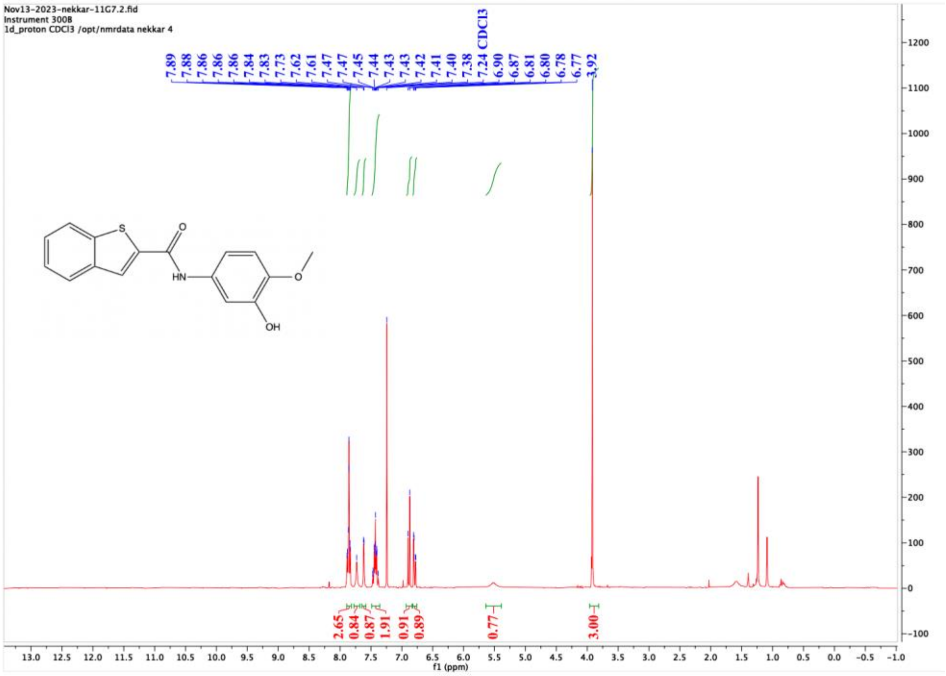

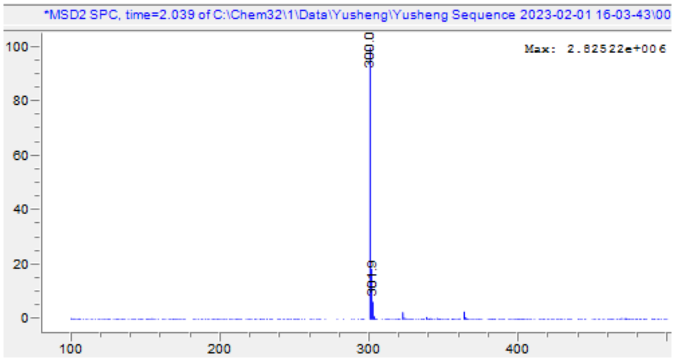

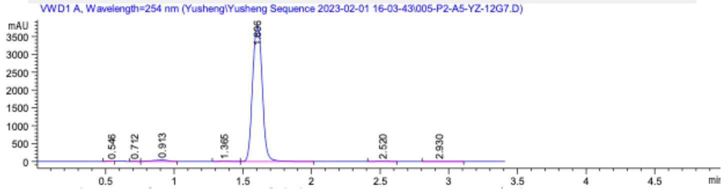

Peak #	RetTime [min]	Type	Width [min]	Area [mAU*s]	Height [mAU]	Area %
1	0.546	BB	0.0595	8.16533	2.09548	0.0432
2	0.712	BB	0.0388	7.64854	3.20799	0.0405
3	0.913	BB	0.1042	268.51862	38.13187	1.4211
4	1.365	BB	0.0621	47.01423	11.65625	0.2488
5	1.606	BB	0.0792	1.85021e4	3776.62402	97.9228
6	2.520	BB	0.0807	48.40391	9.45237	0.2562
7	2.930	BB	0.1038	12.72194	1.83742	0.0673
Totals :				1.88946e4	3843.00540	

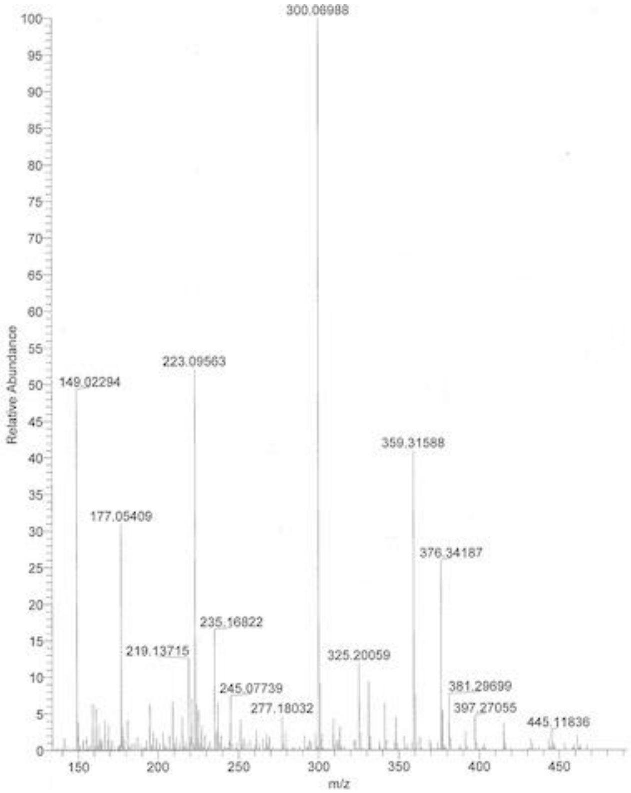
300.06988
223.09563
149.02294
359.31588
177.05409
376.34187
235.16822
219.13715
325.20059
245.07739
381.29699
277.18032
397.27055
445.11836
Relative Abundance
m/z

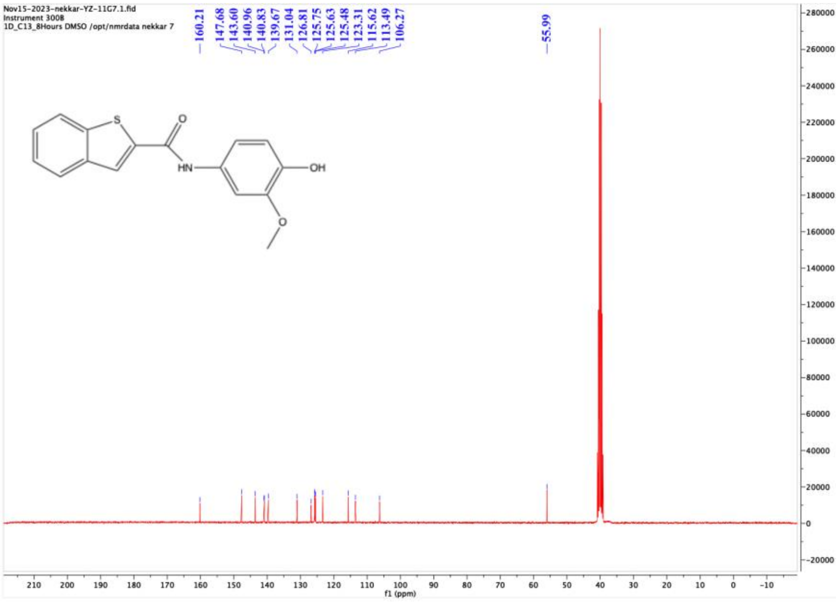
Nov15-2023-nekkar-YZ-11G7.1.fid
Instrument 300B
1D_C13_8Hours DMSO /opt/nmrdata nekkar 7
160.21
147.68
143.60
140.96
140.83
139.67
131.04
126.81
125.75
125.63
125.48
123.31
115.62
113.49
106.27
55.99
S
O
HN
OH
O
f1 (ppm)

7. Analytical data for *N*-(3,4-dimethoxyphenyl)benzo[*b*]thiophene-2-carboxamide (5c)

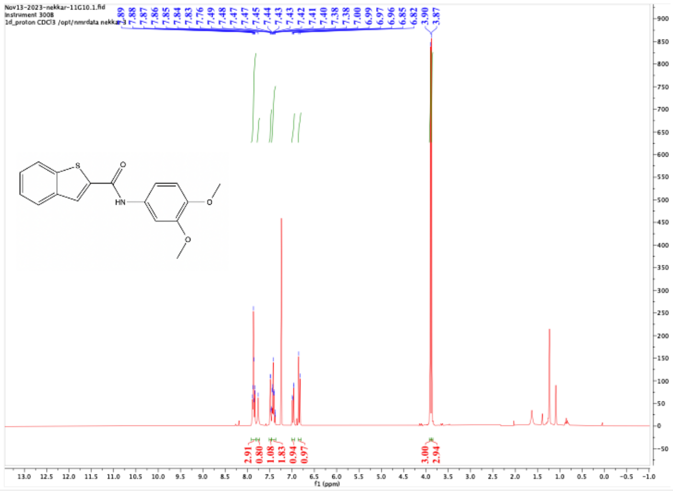

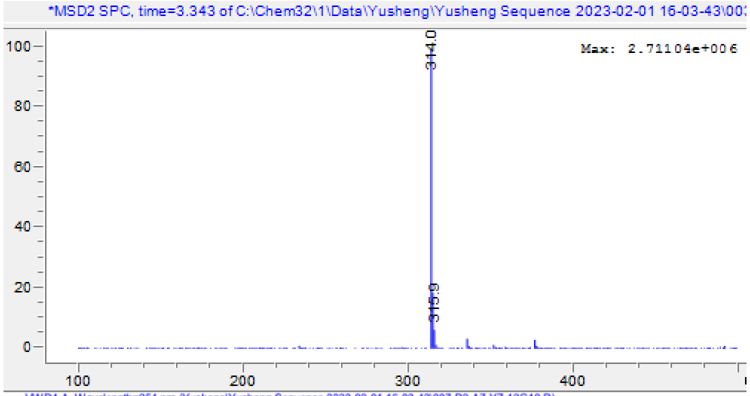

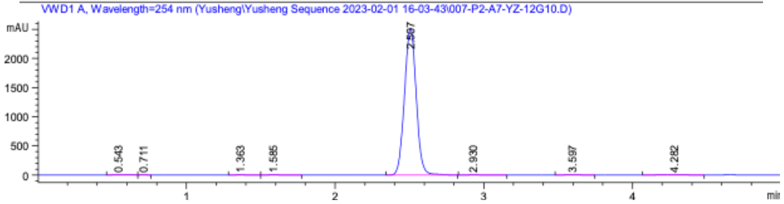

Peak #	RetTime [min]	Type	Width [min]	Area [mAU*s]	Height [mAU]	Area %
1	0.543	BB	0.0916	49.06858	7.28762	0.3526
2	0.711	BB	0.0412	7.97535	3.08173	0.0573
3	1.363	BB	0.0604	17.66060	4.44508	0.1269
4	1.585	BB	0.0661	23.18388	5.29710	0.1666
5	2.507	BB	0.0847	1.37490e4	2516.75195	98.8108
6	2.930	BB	0.1007	13.40386	2.01255	0.0963
7	3.597	BB	0.0999	14.15774	2.26428	0.1017
8	4.282	BB	0.1401	40.02562	4.32154	0.2877
Totals :				1.39145e4	2545.46186	

8. Analytical data for *N*-(4-methoxyphenyl)benzo[*b*]thiophene-2-carboxamide (5d)

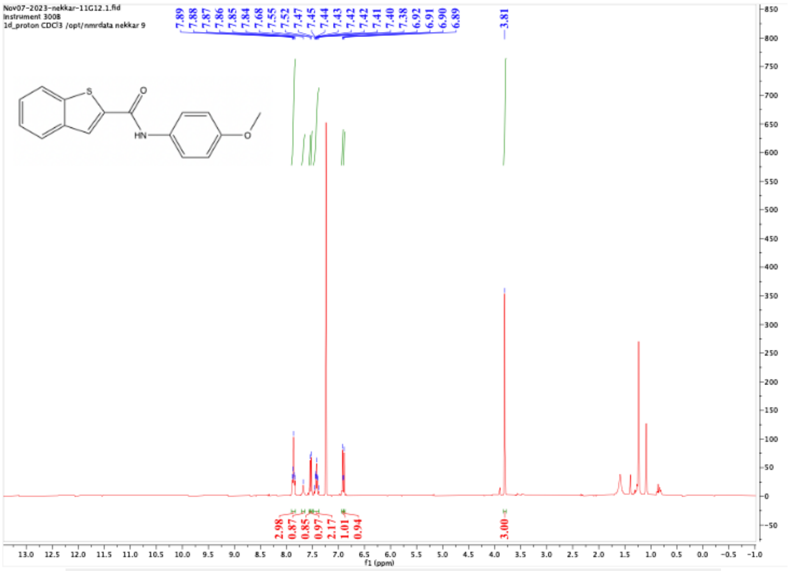

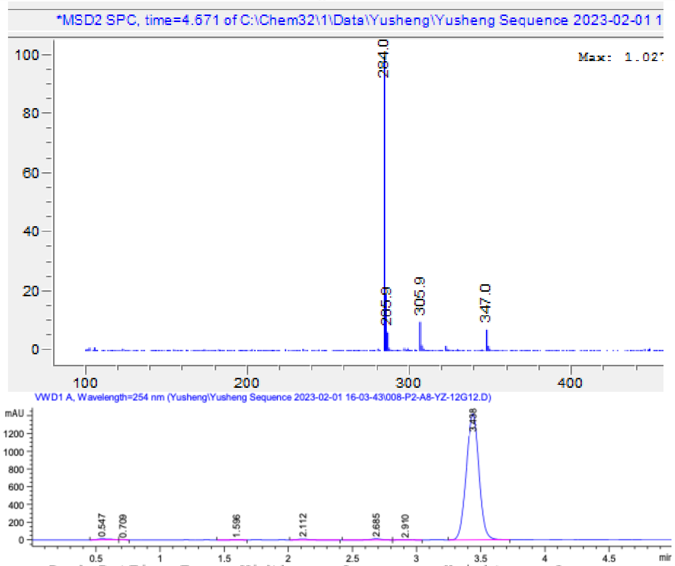

Peak #	RetTime [min]	Type	Width [min]	Area [mAU*s]	Height [mAU]	Area %
1	0.547	BB	0.0972	72.36466	10.52991	0.7274
2	0.709	BB	0.0397	6.78568	2.75368	0.0682
3	1.596	BB	0.0690	9.26006	2.11881	0.0931
4	2.112	BB	0.0763	50.42281	10.26890	0.5068
5	2.685	BB	0.0827	62.03573	11.54520	0.6235
6	2.910	BB	0.0863	13.47597	2.48512	0.1354
7	3.438	BB	0.1069	9734.72559	1421.36951	97.8456

9. ThT-based aggregation kinetics background readings

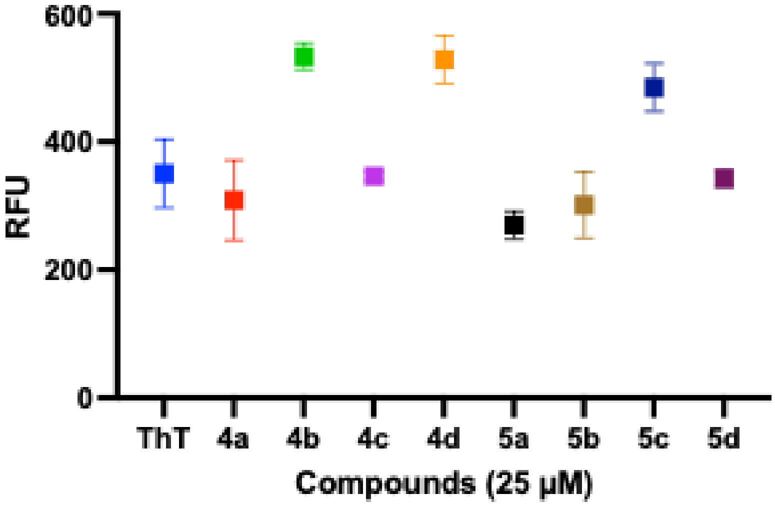

Figure S9. Background readings of compounds **4a–d** and **5a–d** (25 μM each), with ThT in the absence of Aβ42 at 37 °C in phosphate buffer after 24 h incubation. Aggregation kinetics were monitored by ThT at 440 nm excitation and 490 nm emission. Results are averages of three independent experiments in triplicate measurements.

10. Effect of compounds 4a–d and 5a–d on preformed Aβ42 aggregates

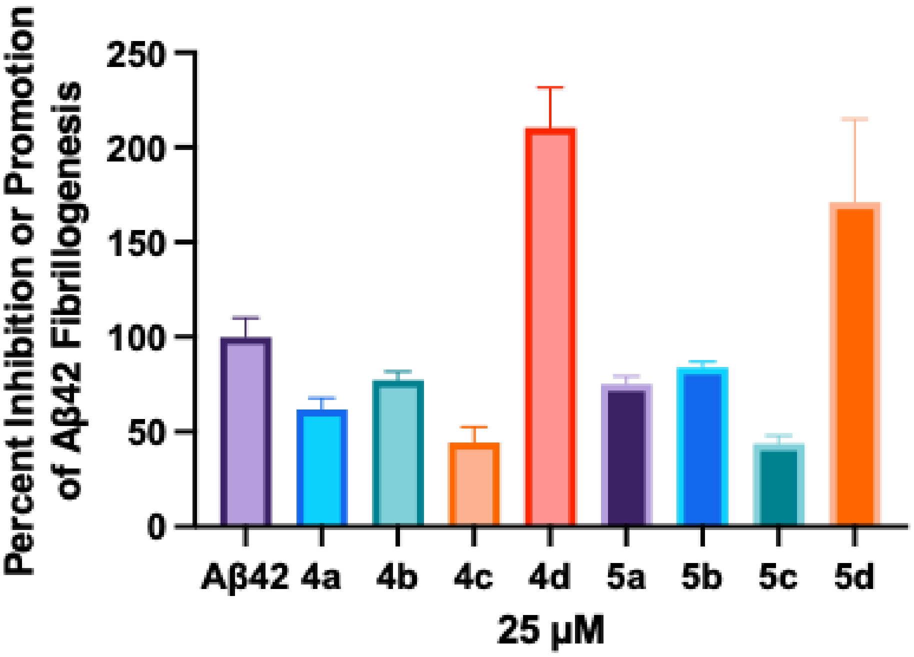

Figure S10. The effect of compounds **4a–d** and **5a–d** (25 μM each) on preformed Aβ42 (10 μM), was determined using ThT fluorescence at 37 °C in phosphate buffer after 24 h incubation. Aggregation kinetics were monitored by ThT (440 nm excitation and 490 nm emission). Results are averages of two independent experiments (n = 3).

11. Congo red UV assay for compounds 4b, 4d, 5b, 5d and resveratrol (RVT)

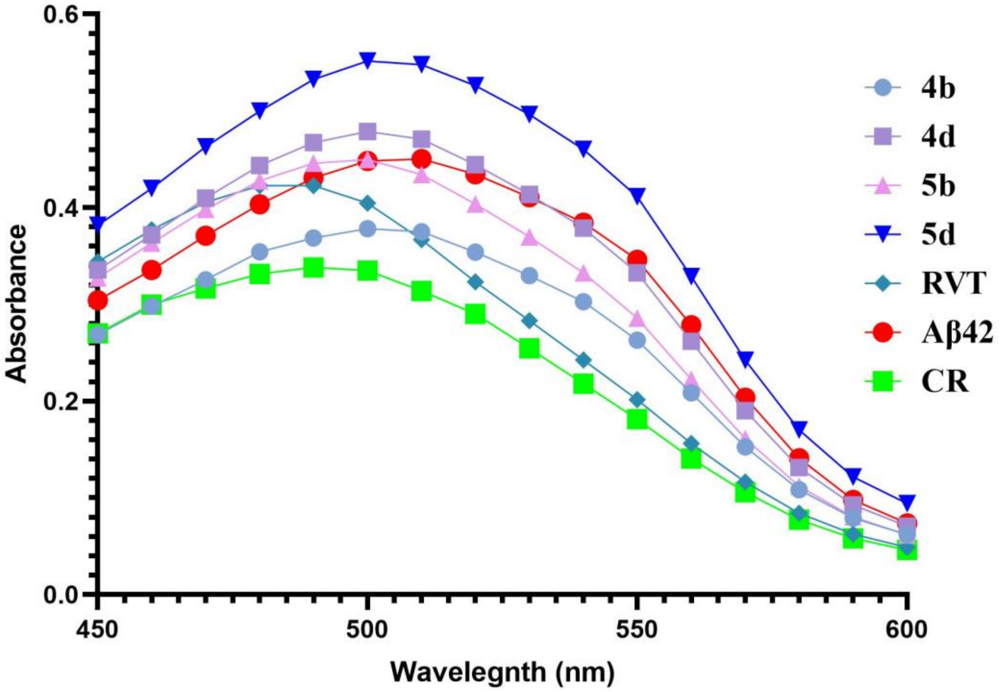

Figure S11. CR-assay curve of Aβ42 (20 μM) in the presence and absence of **4b, 4d, 5b, 5d** and RVT at 25 μM. The CR assay absorbance was measured by UV scanning from 450– 600 nm after 24 h incubation at 37 °C. Results are averages of two independent experiments (n = 3).

12. Table S1. Physicochemical properties of 4a–d and 5a–d calculated using the web tool SwissADME

Compd	Molecular Weight	No. of H-Bond Acceptors	No. of H-Bond Donors	Number of Rotatable Bonds	Polar Surface Area (TPSA) $Å^2$	Consensus Log P	BBB Perm.
**4a**	283.28	4	2	4	71.70	2.54	Yes
**4b**	283.28	4	2	4	71.70	2.50	Yes
**4c**	297.31	4	1	5	60.70	2.88	Yes
**4d**	267.28	3	1	4	51.47	2.94	Yes
**5a**	299.34	3	2	4	86.80	3.26	Yes
**5b**	299.34	3	2	4	86.80	3.29	Yes
**5c**	313.37	3	1	5	75.80	3.62	Yes
**5d**	283.35	2	1	4	66.57	3.61	Yes
